# Talaromarins A–F: Six New Isocoumarins from Mangrove-Derived Fungus *Talaromyces flavus* TGGP35

**DOI:** 10.3390/md20060361

**Published:** 2022-05-27

**Authors:** Jin Cai, Xiao-Chen Zhu, Wei-Nv Zeng, Bin Wang, You-Ping Luo, Jing Liu, Min-Jing Chen, Gao-Yu Li, Guo-Lei Huang, Guang-Ying Chen, Jing Xu, Cai-Juan Zheng

**Affiliations:** 1 Key Laboratory of Tropical Medicinal Resource Chemistry of Ministry of Education, College of Chemistry and Chemical Engineering, Hainan Normal University, Haikou 571158, China; caijin20210207@163.com (J.C.); z17889949296@163.com (W.-N.Z.); 15180328507@163.com (B.W.); lyp188089688692022@163.com (Y.-P.L.); liujing3182799099@163.com (J.L.); cmj19151835730@163.com (M.-J.C.); qqwbma_999@163.com (G.-Y.L.); 070333@hainnu.edu.cn (G.-L.H.); chgying@163.com (G.-Y.C.); 2 Key Laboratory of Tropical Medicinal Plant Chemistry of Hainan Province, Haikou 571158, China; 3 Key Laboratory of Advanced Materials of Tropical Island Resources of Ministry of Education, School of Chemical Engineering and Technology, Hainan University, Haikou 570228, China; zhuxiaochenlucky@163.com

**Keywords:** *Talaromyces flavus*, isocoumarins, antioxidant, antibacterial, *α*-glucosidase inhibitory activity, anti-phytopathogenic

## Abstract

Six new isocoumarin derivative talaromarins A-F (**1**–**6**), along with 17 known analogues (**7**–**23**), were isolated from the mangrove-derived fungus *Talaromyces flavus* (Eurotiales: Trichocomaceae) TGGP35. Their structures were identified by detailed IR, UV, 1D/2D NMR and HR-ESI-MS spectra. The absolute configurations of new compounds were determined by the modified Mosher’s method and a comparison of their CD spectra with dihydroisocoumarins described in the literature. The antioxidant, antibacterial, anti-phytopathogenic and inhibitory activity against α-glucosidase of all the isolated compounds were tested. Compounds **6**–**11**, **17**–**19** and **21**–**22** showed similar or better antioxidant activity than the IC_50_ values ranging from 0.009 to 0.27 mM, compared with the positive control trolox (IC_50_ = 0.29 mM). Compounds **10**, **18**, **21** and **23** exhibited strong inhibitory activities against α-glucosidase with IC_50_ values ranging from 0.10 to 0.62 mM, while the positive control acarbose had an IC_50_ value of 0.5 mM. All compounds showed no antibacterial or anti-phytopathogenic activity at the concentrations of 50 μg/mL and 1 mg/mL, respectively. These results indicated that isocoumarins will be useful to developing antioxidants and as diabetes control agents.

## 1. Introduction

Marine fungi, particularly the mangrove-derived fungi, have proven to be a prolific source of structurally novel and biologically active secondary metabolites, which increasingly attracted the attention of both pharmaceutical and natural product chemists [1,2,3]. Up to now, more than 1400 new secondary metabolites produced by mangrove-derived fungi have been reported, including polyketides, alkaloids, terpenes and so on, and more than 40% of secondary metabolites displayed cytotoxic, antibacterial and insecticidal activities etc. [3,4,5,6,7,8]. Among them, isocoumarins are lactone-type derivatives derived from the polyketide pathway [9,10], and they possess a wide range of pharmacological activities such as antibacterial 7-hydroxyoospolactone and parapholactone [11], anti-inflammatory (±)-prunomarin A [12], cytotoxic lunatinin [13], insecticidal peniciisocoumarins A and B, antiplasmodial monocerin [14], antioxidant and *α*-glucosidase inhibitory penicimarin N [15], brine shrimp (*Artemia salina*) lethal and broad-spectrum antimicrobial penicimarins G–K [16,17], penicoffrazins B and C [18].

The genus of *Talaromyces* has been studied and applied as a biocontrol agent, as a rich source of chitinolytic enzymes and producer of secondary metabolites [19,20,21]. Different classes of bioactive secondary metabolites have been found from the genus of *Talaromyces* [19,20,21], including some bioactive isocoumarins, such as selective antimigratory talarolactone A [22], *α*-glucosidase inhibitory sescandelin B and 3,4-dimethyl-6,8-dihydroxyisocoumarin [23], antibacterial tratenopyrone [24], antibacteria and antifungi (-)-8-hydroxy-3-(4-hydroxypentyl)-3,4-dihydroisocoumarin [25]. These results indicated that the genus of *Talaromyces* can be used for the control of pathogenic bacteria, phytopathogenic microorganisms, diabetes control agents and so on [19,26].

**Figure 1:**
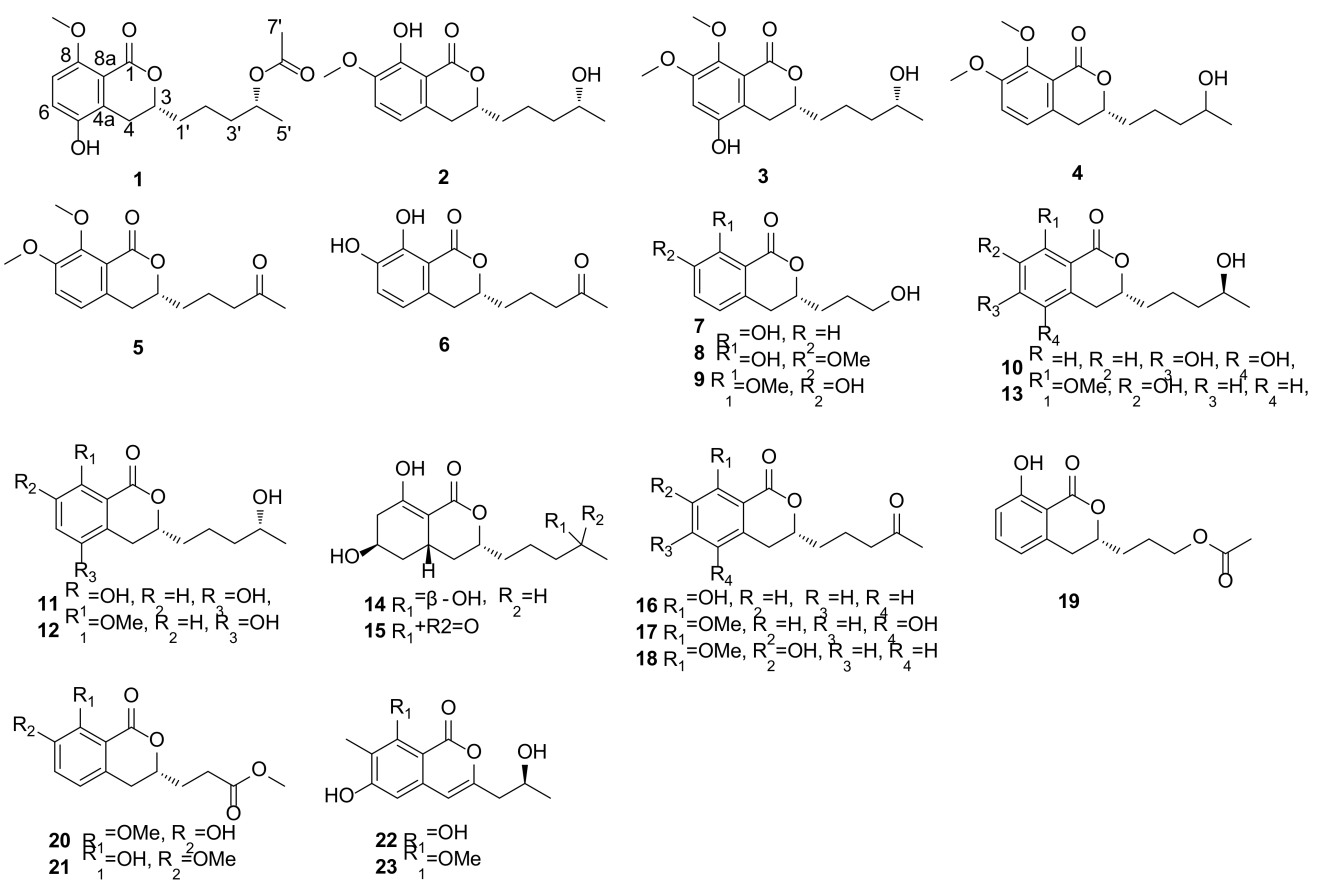
The structures of compounds **1**–**23**.

As part of our continuing exploration of structurally novel and biologically interesting secondary metabolites from mangrove-derived fungi [27,28,29,30,31,32,33], a fungus *Talaromyces flavus* TGGP35 obtained from the medicinal mangrove plant *Acanthus ilicifolius* attracted our attention, because of the EtOAc extract of *T. flavus* TGGP35 showed potent antioxidant activity. A chemical investigation of this fungus led to the identification of six new isocoumarins talaromarins A-F (**1**–**6**) and 17 known analogues (**7**–**23**) (Figure 1). Herein, we report the isolation, structure identification, and bioactivities of these compounds.

## 2. Results and Discussion

**Figure 2:**
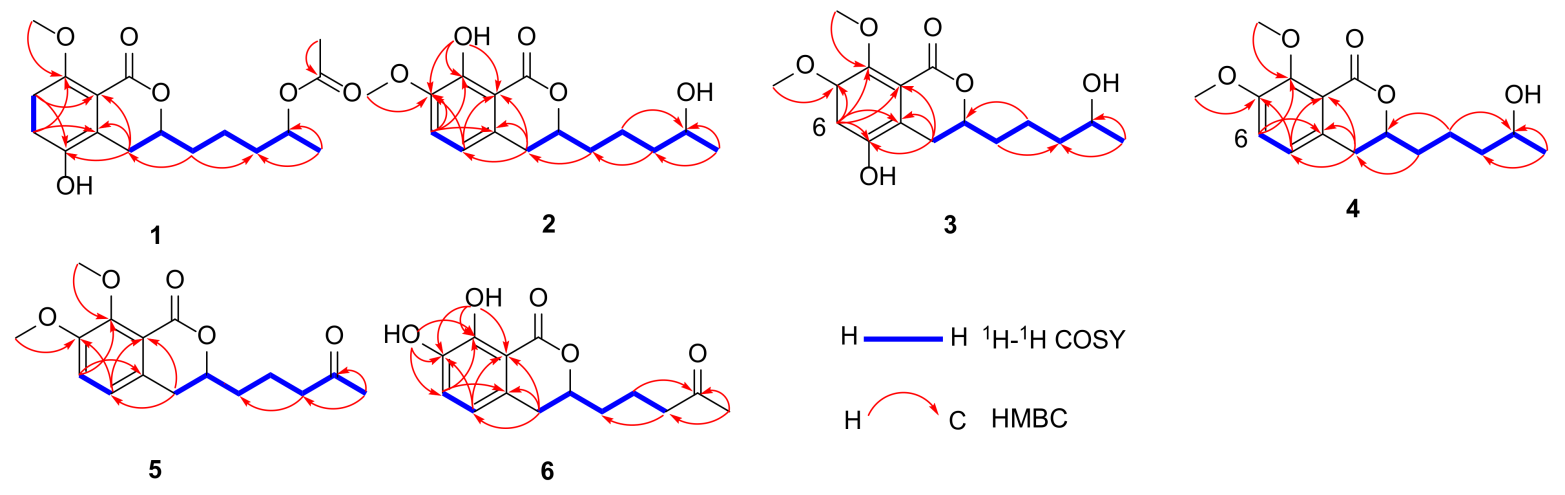
^1^H-^1^H COSY and key HMBC correlations for compounds **1**–**6**.

Compound **1** was isolated as a colorless oil. The molecular formula of **1** was established as C_17_H_2__2_O_6_ (seven degrees of unsaturation) by its HR-ESI-MS spectrum at *m*/*z* 323.1475 [M + H]^+^ (calcd for C_17_H_23_O_6_, 323.1472). The IR spectrum showed the presence of a hydroxyl group (3432 cm^−1^) and an aromatic ring (1743, 1618 and 1388 cm^−1^) in **1**. The ^1^H-NMR data (Table 1) displayed a pair of ortho coupled aromatic protons at *δ*_H_ 7.00 (d, *J* = 8.8 Hz) and 6.78 (d, *J* = 8.8 Hz), one methoxyl group at *δ*_H_ 3.88 (s), two oxygenated methine groups at *δ*_H_ 4.90 (m) and 4.34 (m), four methylene groups at *δ*_H_ [3.08 (m) and 2.64 (m)], 1.85 (m), 1.57 (m) and 1.52 (m), one methyl group at *δ*_H_ 1.22 (d, *J* = 6.4 Hz). The combination of ^13^C NMR and DEPT data (Table 2) exhibited 17 carbon resonances, including two ester carbonyls at *δ*_C_ (171.1 and 163.1), six aromatic carbons at *δ*_C_ (155.5, 145.3, 128.4, 121.2, 114.6 and 111.5), one methoxyl group at *δ*_C_ (56.6), two oxygenated methine groups at *δ*_C_ (77.4 and 70.9), four methylene groups at *δ*_C_ (35.7, 34.7, 28.0 and 21.0), two methyl groups at *δ*_C_ (21.6 and 20.1). The above 1D NMR spectroscopic data indicated that **1** had an isocoumarin skeleton structure, and **1** is similar to penicimarin G (**12**) [16]. The major difference was the presence of an acetoxy group at [(*δ*_C_ 171.1 (C), 21.6 (CH_3_) and *δ*_H_ 2.04, (s)] in **1**, indicating that the hydroxyl group in **12** was replaced by an acetoxy group in **1**. The ^1^H-^1^H COSY correlations of CH_2_(2′)–CH_2_(3′)−CH(4′)−CH_3_(5′), combined with the HMBC correlations from H-5′ to C-4′/C-3′ (Figure 2), confirmed the acetoxy group connected at C-4′ in **1**, and the planar structure of **1** was determined (Figure 1).

**Figure 3:**
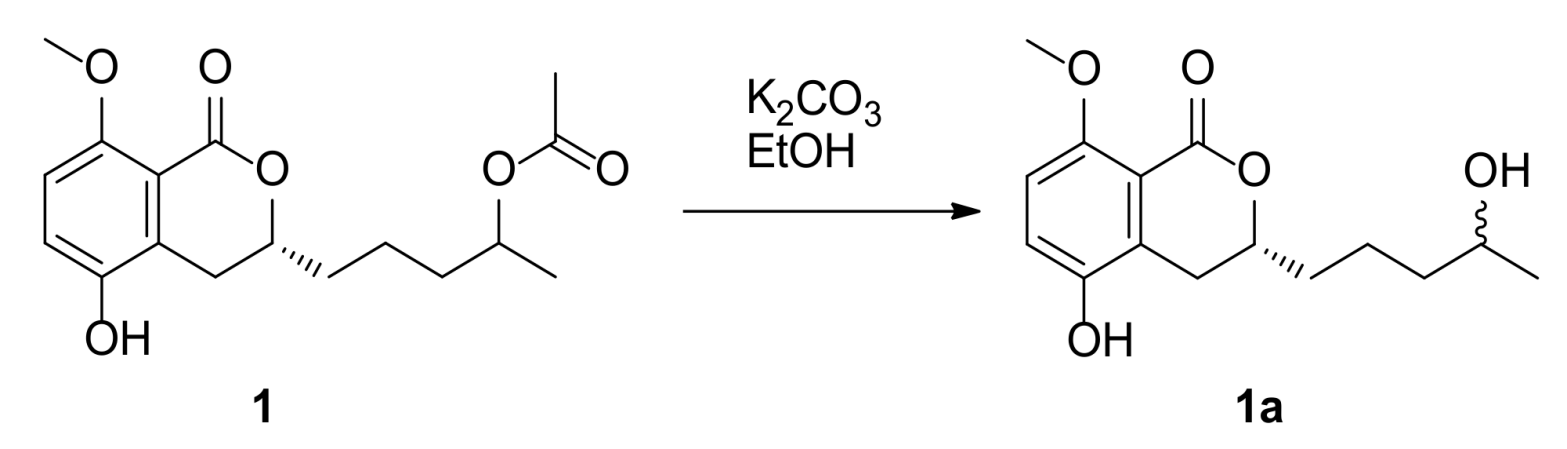
Reaction route of hydrolysis for compound **1**.

**Figure 4:**
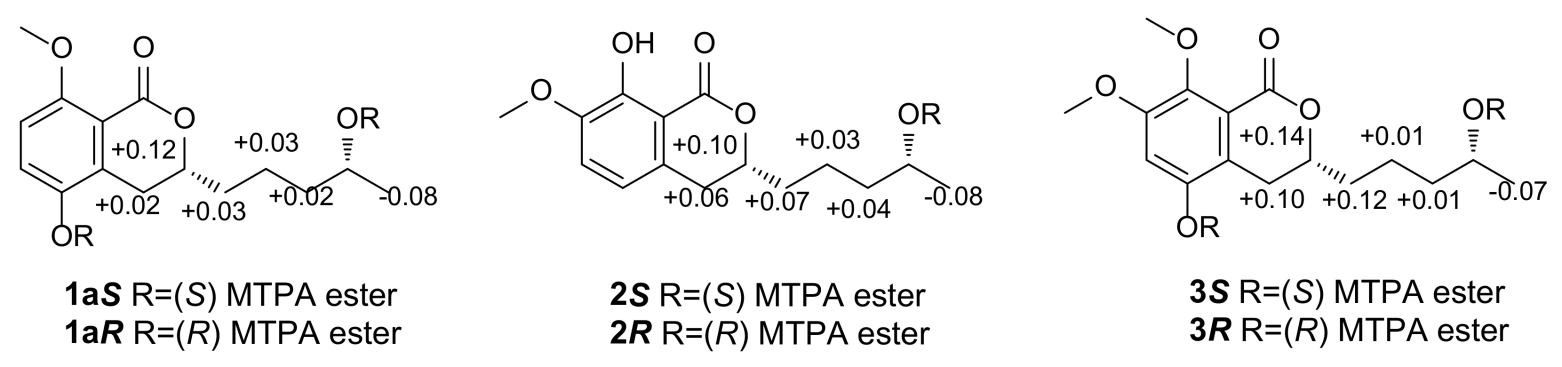
Δ*δ* (=*δ_S_*−*δ_R_*) values for (*S*)- and (*R*)-MTPA esters of compounds **1**–**3**.

**Figure 5:**
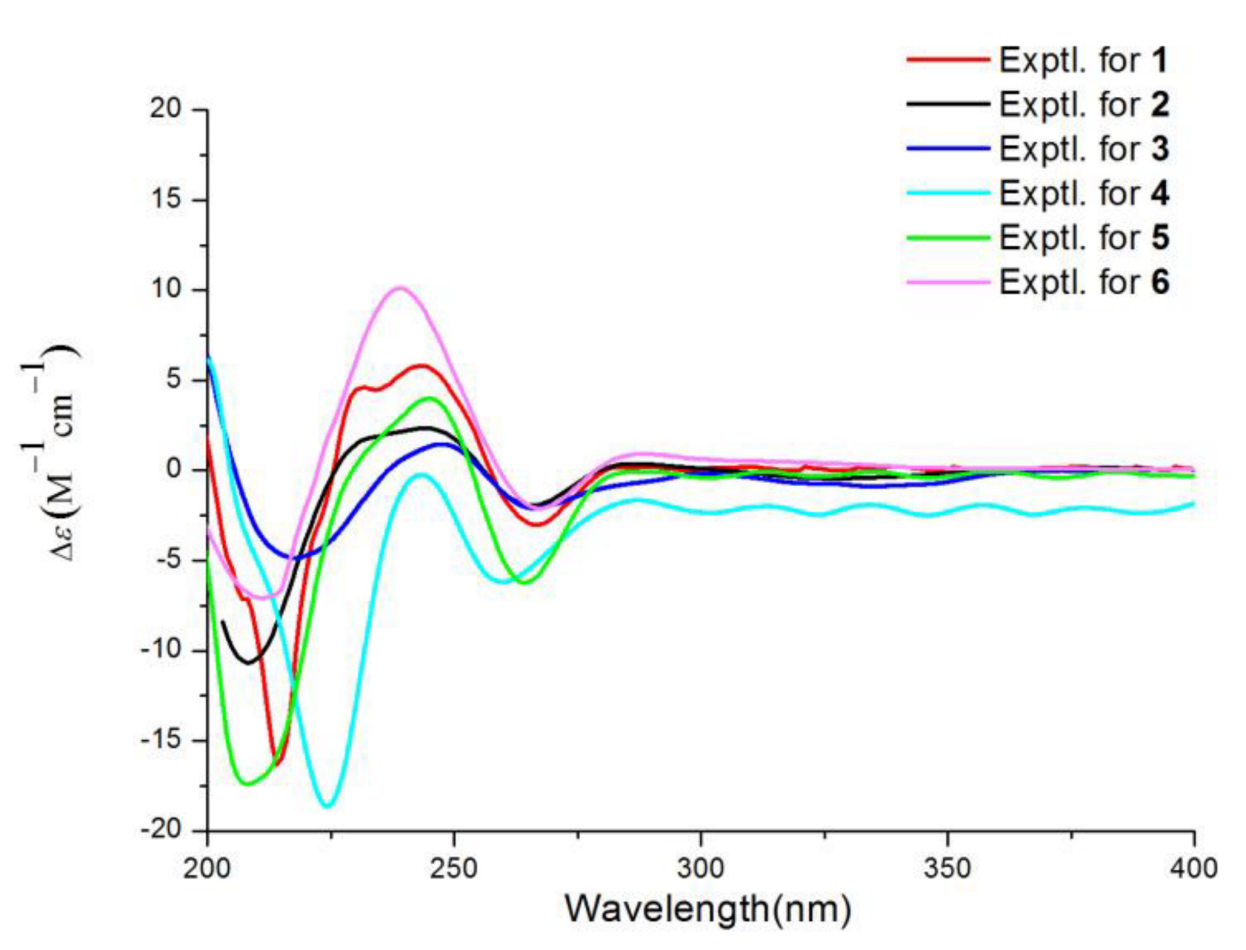
The experimental CD spectra of compounds **1**–**6**.

The absolute configurations of C-3 and C-4′ in **1** were determined by chemical hydrolysis, modified Mosher’s method and a comparison of CD spectra with dihydroisocoumarins described in the literature [28,34]. The major hydrolysis product (**1a**) of **1** was obtained with K_2_CO_3_ and anhydrous ethanol at 28 °C for 1.5 h (Figure 3), and **1a** showed the same planar structure with **12** [16]. The modified Mosher’s method was used to determine the configuration of C-4′ for **1a**. The differences in ^1^H NMR chemical shifts of **1a** between (*S*)- and (*R*)-MTPA esters (Δ*δ* = *δ_S_* − *δ_R_*) were calculated to assign the absolute configuration of C-4′ to be *R* (Figure 4), the same as **12** [16]. The negative cotton effect at 266 nm suggested the *R* configuration at C-3 (Figure 5), by comparison with data for dihydroisocoumarins described in the literature [34]. Thus, the structure of **1** was determined and named talaromarin A.

Compound **2** was isolated as a white powder. The molecular formula was deduced to be C_15_H_20_O_5_ on the basis of HR-ESI-MS spectrum, implying six degrees of unsaturation. According to the IR spectrum, the hydroxyl group (3414 cm^−1^) and aromatic rings (1668, 1619, 1586, 1502 and 1442 cm^−1^) were observed. The ^1^H and ^13^C NMR spectroscopic data (Table 1 and Table 2) revealed that **2** also belonged to the isocoumarin class and had a similar structural relationship to penicimarin M [17], except for the presence of one oxygenated methine at [*δ*_H_ 3.80 (m), *δ*_C_ 67.9 (CH)] for C-4′, and the absence of a carbonyl group at *δ*_C_ 208.4 (C) in **2**. The above results suggested a carbonyl group in penicimarin M was replaced by an oxygenated methine group in **2**. Furthermore, the ^1^H-^1^H COSY correlations of H-5′ to H-4′ and H-3′ to H-2′/H-4′, and the HMBC correlations from the methyl H-5′ to C-3′/C-4′ established the oxygenated methine at C-4′ (Figure 2). The absolute configuration of C-4′ was determined as *R* by Mosher’s method [28] (Figure 4). The negative cotton effect at 265 nm suggested the *R* configuration at C-3 (Figure 5), by comparison with data for dihydroisocoumarins described in the literature [34]. Thus, the absolute configuration of **2** was established as 3*R*,4′*R* and named talaromarin B.

Compound **3** was isolated as a yellow oil, with the molecular formula C_16_H_22_O_6_ (six degrees of unsaturation) by the HR-ESI-MS spectrum. The IR spectrum indicated that **3** had hydroxyl group (3415 cm^−1^) and aromatic ring (1638, 1618 and 1384 cm^−1^). The ^1^H, ^13^C NMR data (Table 1 and Table 2) and HR-ESI-MS data revealed that **3** closely resembled those of **2**, the main differences were the presence of a methoxyl group at [*δ*_H_ 3.89 (s), *δ*_C_ 61.8 (CH_3_)] in **3**, and an aromatic proton signal at *δ*_H_ 6.62 (d, *J* = 8.0 Hz) was absented in **3**. Moreover, the chemical shift of C-5 at *δ*_C_ (117.1) in **2** was downfield-shifted to *δ*_C_ (147.7) in **3**. The HMBC correlations from 8-OCH_3_ to C-8, 7-OCH_3_ to C-7 and H-6 to C-8/C-7/C-4a (Figure 2), indicated the additional methoxyl group was attached to C-8 and the hydroxyl group was connected to C-5 (Figure 2). The absolute configurations of C-3 and C-4′ were determined to be the same *R* by comparison with CD data described in the literature [34] and Mosher’s method [28] (Figure 4 and Figure 5). Thus, the structure of **3** was determined and named talaromarin C.

Compound **4** was isolated as a yellow oil, and the molecular formula was established as C_16_H_22_O_5_ (six degrees of unsaturation) on the basis of its HR-ESI-MS spectrum. The IR spectrum of **4** displayed absorption bands for hydroxyl (3475 cm^−1^), carbonyl (1706 cm^−1^) and aromatic (1637 and 1617 cm^−1^) groups. The ^1^H and ^13^C NMR spectroscopic data (Table 1 and Table 2) suggested that **4** was very similar to those of **2**, the only difference was the presence of a methoxyl group at [*δ*_H_ 3.96 (s) and *δ*_C_ 61.7 (CH_3_)] in **4**. The location of the methoxyl groups at C-7 and C-8 were established by HMBC correlations from 7-OCH_3_ to C-7, 8-OCH_3_ to C-8, H-6 to C-8/C-4a and H-5 to C-7/C-8a/C-4 (Figure 2). The ^1^H-^1^H COSY, HMQC, and HMBC spectra established the complete assignment for **4** (Figure 2). Mosher’s method was tried to determine the absolute configuration of C-4′ in **4** [28]; unfortunately, the reaction failed. The negative cotton effect at 259 nm suggested the *R* configuration at C-3 (Figure 5) [34]. Thus, the structure of **4** was determined and named talaromarin D.

Compound **5** was isolated as a colorless oil and had the molecular formula of C_16_H_20_O_5_ as determined by HR-ESI-MS and NMR data, requiring seven degrees of unsaturation. The presence of an aromatic ring (1638, 1617 cm^−1^) was observed in the IR spectrum. The ^1^H and ^13^C NMR data (Table 1 and Table 2) of **5** were structurally similar to those of **4**, except for the presence of a ketone carbonyl carbon at *δ*_C_ 208.7 (C) and the absence of one oxygenated methine carbon at [*δ*_C_ 68.1 (CH), *δ*_H_ 3.83 (m)] at C-4′ in **5**, indicating that the oxygenated methine group in **4** was replaced by a carbonyl group for C-4′ in **5**. The HMBC correlations from H-3′ to C-1′, H-5′ to C-4′/C-3′ further confirmed **5** with a carbonyl unit at C-4′ (Figure 2). The whole structure was further determined by the 2D NMR spectra (Figure 2). The absolute configuration of C-3 was determined as *R* by CD spectra (Figure 5) [34], and **5** was named talaromarin E.

Compound **6** was isolated as a yellow oil. The molecular formula of **6** was established as C_14_H_16_O_5_ (seven degrees of unsaturation) on the basis of its HR-ESI-MS data. The IR spectrum of **6** showed the hydroxyl group at 3461 and 3407 cm^−1^ and the aromatic rings at 1736 and 1671 cm^−1^. The ^1^H and ^13^C NMR spectroscopic data (Table 1 and Table 2) revealed that **6** was an isocoumarin derivative, with a similar structural relationship to peniciisocoumarin D (**18**), the obvious difference was that **6** lacked a methoxy group at C-8. The methoxy group (8-OMe) in **18** was replaced by a hydroxy group (8-OH) in **6**, which was supported by the appearance of a hydrogen-bonded hydroxyl group at *δ*_H_ 11.00 (s). The HMBC correlations from the hydroxyl group 8-OH to C-8a/C-8/C-7 further confirmed the 8-OH was connected at C-8 (*δ*_C_ 149.1) (Figure 2). The ^1^H-^1^H COSY, HMQC, and HMBC spectra determined the complete assignment for **6** (Figure 2). The absolute configuration of C-3 was determined as *R* by CD spectroscopy (Figure 5) [34] and **6** was named talaromarin F.

By comparing physical and spectroscopic data with the literature, the 17 known homologues were identified as (*R*)-3-(3-hydroxypropyl)-8-hydroxy-3,4-dihydroisocoumarin (**7**) [35], peniciisocoumarin C (**8**) [28], 7-hydroxy-3-(3-hydroxypropyl)-8-methoxyisochroman-1-one (**9**) [34], 5,6-dihydroxy-3-(4-hydroxypentyl)-isochroman-1-one (**10**) [23], peniciisocoumarin F (**11**) [28], penicimarin G (**12**) [16], penicimarin C (**13**) [36], peniciisocoumarin A (**14**) [28], peniciisocoumarin B (**15**) [28], aspergillumarin A (**16**) [37], penicimarin H (**17**) [16], peniciisocoumarin D (**18**) [28], peniciisocoumarin G (**19**) [28], peniciisocoumarin E (**20**) [28], penicimarin N (**21**) [15], 6,8-dihydroxy-3-(2-hydroxypropyl)-7-methyl-1*H*-isochromen-1-one (**22**) [38] and pestalotiorin (**23**) [39].

**Scheme 1:**
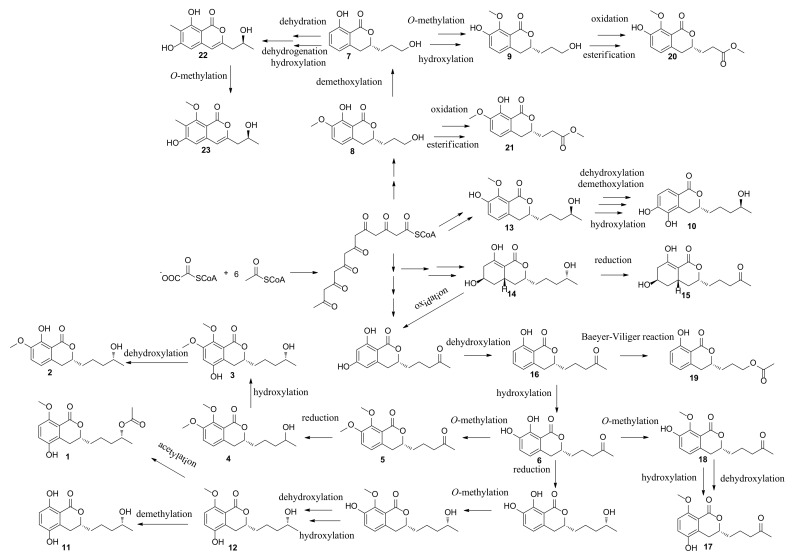
Plausible biosynthetic pathways of compounds **1**–**23**.

The plausible biosynthetic pathways of compounds **1**–**23** were also proposed (Scheme 1). Isocoumarins were originated from the acetate-malonate or the polyketide synthase (PKS) pathway [10]. Peniciisocoumarin C (**8**), penicimarin C (**13**) and peniciisocoumarin A (**14**) would be biosynthesized from malonyl-CoA and acetyl-CoA and can be considered as intermediates which would be transformed to other isolated compounds from the fungus TGGP35. Compound **8** would be transformed to **7**, **9** and **20**–**23** by condensation, aromatization, esterification, dihydroxylation, methoxylation reaction and so on. Compound **13** would be transformed to **10** by dehydroxylation, demethoxylation and dehydroxylation reaction. Compounds **1**–**6**, **10**–**15** and **17**–**19** would be deduced from **14** with dihydroxylation, methoxylation, methylation, hydroxylation, esterification, acetylation, Baeyer–Viliger oxidation reaction, etc.

The antioxidant activities of compounds **1**–**23** were evaluated. Compounds **6**–**11**, **17**–**19** and **22** exhibited potent antioxidant activity with the IC_50_ values ranging from 0.009 to 0.27 mM, while the positive control trolox was IC_50_ = 0.29 mM (Table 3).

The preliminary structure–activity relationship of the isolated isocoumarins was discussed. The substitution site, orientation of hydroxyl and methoxy groups on the skeleton of isocoumarins, and the substitution of different groups by side chain C-4′ can affect their antioxidant activity. Compound **2** which possesses a hydroxyl group on C-8, showed better antioxidant activity than that of **3** and **4**, indicating that the chelated hydroxyl group at C-8 is important in enhancing antioxidant activity. Compound **6** possesses two hydroxyl groups at C-7 and C-8, which showed higher antioxidant activity than **16** (only one hydroxyl group on C-8), indicating that the hydroxyl group at C-7 is an important antioxidant activity site. Compounds **17** and **18** possess a ketone group at C-4′, which showed higher antioxidant activity than **12** and **13,** which have an oxygenated methine at C-4′, suggesting that the substitution of different groups by side chain at C-4′ can affect antioxidant activity. Compound **22** possesses a hydroxyl group at C-8, showed higher antioxidant activity than **23**, suggesting that the chelated hydroxyl group at C-8 is important in enhancing antioxidant activity. Furthermore, compounds **7**, **8**, **19** and **21** show antioxidant activities, which may be due to the existence of a chelated hydroxyl group.

Compounds **10**, **18**, **21**, and **23** showed strong inhibitory activity against *α*-glucosidase with the IC_50_ values of 0.10, 0.38, 0.62 and 0.54 mM, respectively (the positive control acarbose with the IC_50_ value of 0.5 mM).

All compounds were tested for their antibacterial activities against *Staphylococcus. aureus*, *Escherichia coli*, *S. epidermidis and Pseudomonas aeruginosa*; however, all compounds showed no antibacterial activity at the concentration of 50 μg/mL. All compounds showed no biological activity against five phytopathogens (*Colletotrichum asianum*, *C. acutatum*, *Fusarium oxysporum*, *Pyricularia oryzae* and *Curvularia australiensis*) at the concentration of 1 mg/mL.

## 3. Materials and Methods

### 3.1. General Experimental Procedures

Optical rotations were measured on a JASCO P-1020 digital polarimeter (JASCO, Tokyo, Japan). IR spectra were recorded on a Thermo Nicolet 6700 (using KBr disks) spectrophotometer. UV spectra were measured on a PERSEE TU-1990 spectrophotometer. CD spectra were recorded on a Mos-450 spectrometer and 1D and 2D NMR spectra were recorded on a Bruker AV spectrometer (400 MH_Z_ for ^1^H and 100 MH_Z_ for ^13^C) and a JNM-ECZS spectrometer (600 HM_Z_ for ^1^H and 125 MH_Z_ for ^13^C). HR-ESI-MS spectra were obtained on a Q-TOF Ultima Global GAA076 LC mass spectrometer. ESI-MS spectra were recorded on a MAT-95-MS mass spectrometer. HPLC were used for the Agilent 1100 prep-HPLC system with an Agilent C18 analytical (9.4 × 250 mm, 5 μm) HPLC column. Silical gel (200−300 mesh, Qingdao Marine Chemical Factory, Qingdao, China) were used for column chromatography (CC). Sephadex LH-20 gel column (GE Healthcare, Bio-Sciences Corp, Piscataway, NJ, USA) were used for CC. Biological activities were tested on an ultra-clean workbench (Suzhou Sujing Company, Suzhou, China) and the biological activities’ results were tested with a full wavelength multifunctional microplate reader (Bio-Tek Instruments, Winooski, VT, USA). Methanol, ethyl acetate, petroleum ether, chloroform, dimethyl sulfoxide and other conventional chemical reagents were used in the experimental operation (Guangzhou Xilong Chemical Reagent Factory, Guangzhou, China).

### 3.2. Fungal Materials

The fungus TGGP35 was isolated from the stem of the mangrove plant *Acanthus ilicifolius*, which were collected in the Dongzhai Port, Haikou, Hainan Province in August, 2015. The fungus was identified according to its morphological characteristics and a molecular biological protocol by 18S rRNA amplification and sequencing of the ITS region. The sequence data have been submitted to GeneBank, with accession number MT071116, and the fungal strain was identified as *Talaromyces flavus* (Eurotiales: Trichocomaceae). The strains have been stored in the Key Laboratory of Tropical Medicinal Resources Chemistry of the Ministry of Education, School of Chemistry and Chemical Engineering, Hainan Normal University (PDA medium, stored at 4 °C).

### 3.3. Fermentation, Extraction and Isolation

The seed culture was prepared in potato liquid medium (6 g sea salt and 10 g peptone in 2 L of potato infusion, in 1 L × 4 erlenmeyer flasks each containing 500 mL seed medium), and incubated on a rotary shaker (170 rpm) for 4 days at 28 °C. In total, 20 mL seed culture was then transferred into 1 L erlenmeyer flasks with solid rice medium with a total of 100 bottles of fermentation (each flask contained 60 mL rice, 0.6 g sea salt and 1.0 g peptone) at 28 °C for 4 weeks. The whole rice fermented material was extracted three times with EtOAc, and then concentrated in vacuo to yield crude extracts (120.8 g). The total crude extracts were subjected to silica gel column chromatography (CC) eluted with petroleum ether/EtOAc (*v*/*v*, gradient 100:0–0:100) and EtOAc/MeOH (*v*/*v*, gradient 100:0–70:30) to generate fifteen fractions (Fr. A-Fr. L). Fr. F (20.5 g) was fractionated by silica gel CC (200-300 mesh) using a gradient elution of petroleum ether/EtOAc system (7:1–0:1) to obtain ten fractions (Fr. F1-Fr. F10) by the TLC analysis. Fr. F2 (10.3 g) was subjected to Sephadex LH-20 (Petroleum ether-CHCl_3_-MeOH, 2:1:1, *v*/*v*), and then to semi-preparative HPLC (MeOH-H_2_O, 70:30, *v*/*v*) to give compounds **1** (4.2 mg), **2** (5.5 mg), **3** (3.8 mg), **5** (4.5 mg) and **7** (4.7 mg). Subfraction Fr. F3 was further separated by semi-preparative HPLC (MeOH-H_2_O, 60:40, *v*/*v*) for four subfractions Fr. F3a-3d. Compounds **6** (4.7 mg), **8** (5.5 mg), **9** (5.8 mg), and **10** (3.5 mg) were isolated from subfraction Fr. F3a. Compounds **11** (3.7 mg), **14** (4.7 mg), **16** (5.5 mg), **17** (5.8 mg) and **19** (3.5 mg) for subfraction Fr. F3b. Compounds **12** (6.3 mg), **13** (3.2 mg) and **15** (5.7 mg) for subfraction Fr. F3c, and compounds **18** (6.8 mg), **19** (6.2 mg) and **21** (3.6 mg) for subfraction Fr. F3d. Fr. F4 (5.4 g) were subjected to Sephadex LH-20 (CHCl_3_-MeOH, 1:1, *v*/*v*), then used for semi-preparative HPLC (MeOH-H_2_O, 65:35, *v*/*v*) to give compound **20** (3.0 mg). Fr. F5 (2.4 g) was subjected to Sephadex LH-20 (CHCl_3_-MeOH, 1:1, *v*/*v*), then used for semi-preparative HPLC (MeOH-H_2_O, 35:65, *v*/*v*) to give compounds **22** (3.4 mg) and **23** (2.7 mg).

*Talaromarin A* (**1**): yellow oil; [*α*]^25^_D_ −21.4 (*c* 0.10, MeOH); UV (MeOH) *λ*_max_ (log *ε*) 330, 221 nm; IR (KBr) *ν*_max_ 3432, 1716, 1618, 1383 cm^−1^; CD (*c* 0.05, MeOH) *λ*_max_ (Δ*ε*) 243 (+5.68), 266 (−2.89) nm; ^1^H and ^13^C NMR data see Table 1 and Table 2; HR-ESI-MS *m*/*z*: 323.1475 [M + H]^+^, (C_17_H_23_O_6_, calcd. for 323.1472).

*Talaromarin B* (**2**): white powder; [*α*]^25^_D_ −23.6 (*c* 0.10, MeOH); UV (MeOH) *λ*_max_ (log *ε*) 330, 255, 223 nm; IR (KBr) *ν*_max_ 3414, 1668, 1619, 1586, 1502, 1442 cm^−1^; CD (*c* 0.05, MeOH) *λ*_max_ (Δ*ε*) 244 (+2.27), 265 (−2.17) nm; ^1^H and ^13^C NMR data see Table 1 and Table 2; HR-ESI-MS *m*/*z*: 279.1240 [M − H]^−^, (C_15_H_19_O_5_, calcd. for 279.1239).

*TalaromarinC* (**3**): yellow oil; [*α*]^25^_D_ −22.0 (*c* 0.10, MeOH); UV (MeOH) *λ*_max_ (log *ε*) 317, 215 nm; IR (KBr) *ν*_max_ 3415, 1638, 1618, 1384 cm^−1^; CD (*c* 0.05, MeOH) *λ*_max_ (Δ*ε*) 248 (+1.36), 266 (−2.01) nm; ^1^H and ^13^C NMR data see Table 1 and Table 2; HR-ESI-MS *m*/*z*: 311.1485 [M + H]^+^, (C_16_H_23_O_6_, calcd. for 311.1482).

*Talaromarin D* (**4**): yellow oil; [*α*]^25^_D_ −21.8 (*c* 0.10, MeOH); UV (MeOH) *λ*_max_ (log *ε*) 313, 222 nm; IR (KBr) *ν*_max_ 3475, 1706, 1637, 1617 cm^−1^; CD (*c* 0.05, MeOH) *λ*_max_ (Δ*ε*) 243 (−0.31), 259 (−6.25) nm; ^1^H and ^13^C NMR data see Table 1 and Table 2; HR-ESI-MS *m*/*z*: 295.1527 [M + H]^+^, (C_16_H_23_O_5_, calcd. for 295.1524).

*Talaromarin E* (**5**): colorless oil; [*α*]^25^_D_ −24.4 (*c* 0.10, MeOH); UV (MeOH) *λ*_max_ (log *ε*) 314, 219 nm; IR (KBr) *ν*_max_ 1638,1617 cm^−1^; CD (*c* 0.05, MeOH) *λ*_max_ (Δ*ε*) 245 (+3.93), 264 (−11.89) nm; ^1^H and ^13^C NMR data see Table 1 and Table 2; HR-ESI-MS *m*/*z*: 293.1370 [M + H]^+^, (C_16_H_21_O_5_, calcd. for 293.1374).

*Talaromarin F* (**6**): yellow oil; [*α*]^25^_D_ −21.2 (*c* 0.10, MeOH); UV (MeOH) *λ*_max_ (log *ε*) 328, 254, 223 nm; IR (KBr) *ν*_max_ 3481, 3407, 1736, 1671 cm^−1^; CD (*c* 0.05, MeOH) *λ*_max_ (Δ*ε*) 238 (+10.12), 267 (−2.07) nm; ^1^H and ^13^C NMR data see Table 1 and Table 2; HR-ESI-MS *m*/*z*: 265.1050 [M + H]^+^, (C_14_H_17_O_5_, calcd. for 265.1053).

### 3.4. Preparations of the (S)- and (R)-MTPA Esters of Compounds ***1***, ***2*** and ***3***

Compound **1** was hydrolyzed in anhydrous ethanol solution for 90 min with potassium carbonate in an equivalent ratio of 1:2; the mixed product after hydrolysis was purified by semi-preparative HPLC (MeOH-H_2_O, 70:30, *v*/*v*) to obtain **1a**. The preparation of (*S*)- and (*R*)-MTPA ester derivatives of **1a**, **2** and **3** was performed as described previously [28].

Hydrolysate of **1** (**1a**): ^1^H NMR (CDCl_3_, 400 MHz): *δ*_H_ 6.99 (1H, d, *J* = 8.8 Hz H-6), 6.79 (1H, d, *J* = 8.8 Hz, H-7), 4.26 (1H, m, H-3), 3.83 (1H, m, H-4′), 3.10 and 2.63 (2H, m, H-4), 1.90 (2H, m, H-1′), 1.70 (2H, m, H-2′), 1.50 (2H, m, H-3′), 1.21 (3H, d, *J* = 6.0 Hz, H-5′); ^13^C NMR (CDCl_3_, 100 MHz): *δ*_C_ 163.3, 159.4, 151.5, 145.1, 135.0, 128.5, 121.2, 111.4, 68.2, 56.6, 39.1, 34.9, 28.0, 23.8, 21.4; ESI-MS *m*/*z* 281.1 [M + H]^+^.

(*S*)-MTPA ester of **1a**: ^1^H NMR (CDCl_3_, 600 MHz): *δ*_H_ 7.29 (1H, d, *J* = 9.0 Hz, H-6), 6.94 (1H, d, *J* = 9.0 Hz, H-7), 5.12 (1H, m, H-4′), 4.21 (1H, m, H-3), 2.64 and 2.49 (2H, m, H-4), 1.86 (2H, m, H-1′), 1.67 (2H, m, H-2′), 1.55 (2H, m, H-3′), 1.25 (3H, d, *J* = 6.6 Hz, H-5′); ESI-MS *m*/*z* 758.2 [M + Na]^+^.

(*R*)-MTPA ester of **1a**: ^1^H NMR (CDCl_3_, 600 MHz): *δ*_H_ 7.31 (1H, d, *J* = 9.0 Hz, H-6), 6.94 (1H, d, *J* = 9.0 Hz, H-7), 5.11 (1H, m, H-4′), 4.09 (1H, m, H-3), 2.62 and 2.41 (2H, m, H-4), 1.83 (2H, m, H-1′), 1.64 (2H, m, H-2′), 1.53 (2H, m, H-3′), 1.33 (3H, d, *J* = 6.0 Hz, H-5′); ESI-MS *m*/*z* 758.2 [M + Na]^+^.

(*S*)-MTPA ester of **2**: ^1^H NMR (CDCl_3_, 600 MHz): *δ*_H_ 7.01 (1H, d, *J* = 7.8 Hz, H-5), 6.63 (1H, d, *J* = 7.8 Hz, H-6), 5.17 (1H, m, H-4′), 4.52 (1H, m, H-3), 2.82 (2H, m, H-4), 1.88 (2H, m, H-1′), 1.62 (2H, m, H-2′), 1.50 (2H, m, H-3′), 1.29 (3H, d, *J* = 6.0 Hz, H-5′); ESI-MS *m*/*z* 758.3 [M + Na]^+^.

(*R*)-MTPA ester of **2**: ^1^H NMR (CDCl_3_, 600 MHz): *δ*_H_ 7.01 (1H, d, *J* = 8.4 Hz, H-5), 6.62 (1H, d, *J* = 8.4 Hz, H-6), 5.16 (1H, m, H-4′), 4.42 (1H, m, H-3), 2.76 (2H, m, H-4), 1.81 (2H, m, H-1′), 1.59 (2H, m, H-2′), 1.46 (2H, m, H-3′), 1.37 (3H, d, *J* = 6.0 Hz, H-5′); ESI-MS *m*/*z* 774.3 [M + K]^+^.

(*S*)-MTPA ester of **3**: ^1^H NMR (CDCl_3_, 600 MHz): *δ*_H_ 6.88 (1H, s, H-6), 5.14 (1H, m, H-4′), 4.21 (1H, m, H-3), 2.43 (2H, m, H-4), 1.72 (2H, m, H-1′), 1.52 (2H, m, H-2′), 1.43 (2H, m, H-3′), 1.35 (3H, d, *J* = 9.4 Hz, H-5′); ESI-MS *m*/*z* 766.6 [M + Na]^+^.

(*R*)-MTPA ester of **3**: ^1^H NMR (CDCl_3_, 600 MHz): *δ*_H_ 6.86 (1H, s, H-6), 5.13 (1H, m, H-4′), 4.07 (1H, m, H-3), 2.33 (2H, m, H-4), 1.65 (2H, m, H-1′), 1.51 (2H, m, H-2′), 1.42 (2H, m, H-3′), 1.28 (3H, d, *J* = 9.4 Hz, H-5′); ESI-MS *m*/*z* 766.0 [M + Na]^+^.

### 3.5. Biological Assays

#### 3.5.1. Antioxidant Activity

The antioxidant activity assay was based on the reported methods [15]. The assay was performed on a 96-well microplate, the reaction was initiated by adding 10 µL of sample solution to 200 µL of ABTS working solution. All test group gradients (including positive control) were of 2.0, 1.0, 0.5, 0.25 mg/mL, respectively. PBS buffer was used as the blank control, DMSO as the negative control, and trolox as the positive control (IC_50_ = 0.29 mM). The antioxidant effect was evaluated by a full wavelength multifunctional microplate reader measurement at 734 nm. The inhibition rate of each sample was calculated according to the following formula: inhibition rate = [(A_blank_ − A_compound_)/A_blank_] × 100%. Finally, the SPSS software was used to calculate the IC_50_ value.

#### 3.5.2. Antibacterial Activity

All compounds were determined against four pathogenic bacteria: *Staphylococcus aureus* (ATCC 25923), *Escherichia coli* (ATCC 25922), *S. epidermidis* (ATCC 17749) and *Pseudomonas aeruginosa* (ATCC 17749). The concentration value of the test group and positive control was 1mg/mL by the microplate assay method [28]. The antibacterial effect was evaluated by a full wavelength multifunctional microplate reader measurement at 630 nm; the broth medium containing pathogenic bacteria was used as the blank group and DMSO as the negative control, ciprofloxacin was used as the positive control. The positive control ciprofloxacin showed antibacterial activities against four pathogenic bacteria *S. aureus*, *E. coil*, *S. epidermidis* and *P. aeruginosa* with the MIC values of 0.097, 0.78, 0.195 and 0.78 μg/mL, respectively.

#### 3.5.3. Anti-Phytopathogenic Activity

All compounds were tested against five plant pathogens (*Colletotrichum asianum*, *C. acutatum*, *Fusarium oxysporum*, *Pyricularia oryzae* and *Curvularia australiensis*) by disk method [40]. DMSO was used as a negative control, carbendazim as a positive control. The concentration values of all test groups, negative control and positive control were 1 mg/mL; the anti-phytopathogenic results were recorded on a vernier caliper.

#### 3.5.4. Inhibitory Activity against α-Glucosidase

The *α*-glucosidase inhibitory activity of the tested compounds was determined using the method in [17], with modifications for carrying it out in 96-well plates. The initial concentration of all test samples (including positive control and negative control) was 1 mg/mL, the optimized method was a mixture of 0.1 mM potassium phosphate buffer (pH = 6.8, 0.5 mL) and 10 mg/L *α*-glucosidase (100 µL), the testing sample (0.5 mL) was incubated at 37 °C for 5 min, and the 2.5 mM (4-nitrophenyl-*β*-*D*-glucopyranoside) PNPG (0.5 mL) was added, followed by mixing. The reaction was carried out at 37 °C for 15 min and then stopped by adding 0.2 M solution of Na_2_CO_3_ (0.75 mL). The inhibitory activity against α-Glucosidase was evaluated by a full wavelength multifunctional microplate reader measurement at 405 nm. Finally, inhibition rate = [(A_control_ − A_compound_)/A_control_] × 100%. The SPSS software was used to calculate the IC_50_ value. DMSO was used as the negative control and acarbose was used as the positive control (IC_50_ = 0.5 mM).

## 4. Conclusions

In summary, 23 secondary metabolites, including six new isocoumarin derivative talaromarins A-F (**1**–**6**) and 17 known analogues (**7**–**23**) were obtained from the mangrove-derived fungus *Talaromyces flavus* TGGP35. Compounds **6**–**11**, **17**–**19** and **22** exerted similar or better antioxidant activity than the positive control trolox (IC_50_ = 0.29 mM), with IC_50_ values ranging from 0.009 to 0.27 mM. Compounds **10**, **18**, **21** and **23** exhibited strong inhibitory activities against *α*-glucosidase with IC_50_ values ranging from 0.10 to 0.62 mM, while the IC_50_ value of positive control acarbose was 0.5 mM. All compounds showed no antibacterial or anti-phytopathogenic activity at the concentrations of 50 μg/mL and 1 mg/mL, respectively. Their plausible biosynthetic pathway and structure–activity relationships were also explored. Therefore, these findings demonstrate the potential of these active compounds as lead compounds for developing antioxidants and as diabetes control agents.

## Figures and Tables

**Table 1:** 1H NMR spectroscopic data (400/600 MHz) (*δ* in ppm, *J* in Hz) for **1**–**6** in CDCl_3_.

Position	1	2	3	4	5	6
3	4.34, m	4.54, m	4.35, m	4.36, m	4.37, m	4.58, m
4	2.64, dd (16.4, 11.6) 3.08, dd (16.4, 2.8)	2.86, m	2.56, m 2.98, m	2.82, m	2.82, m	2.87, m
5		6.62, d (8.0)		6.91, d (8.0)	6.91, d (8.4)	6.61, d (8.0)
6	7.00, d (8.8)	6.98, d (8.4)	6.67, s	7.06, d (8.0)	7.06, d (8.0)	7.07, d (8.0)
7	6.78, d (8.8)					
7-OH						5.53, s
7-OMe		3.87, s	3.84, s	3.88, s	3.88, s	
8-OMe	3.88, s		3.89, s	3.96, s	3.95, s	
8-OH		11.18, s				11.00, s
1′	1.85, m	1.87, m	1.85, m	1.88, m	1.82, m	1.78, m
2′	1.57, m	1.58, m	1.60, m	1.58, m	1.78, m	1.80, m
3′	1.52, m	1.47, m	1.50, m	1.51, m	2.52, t (6.4)	2.53, m
4′	4.90, m	3.80, m	3.82, m	3.83, m		
5′	1.22, d (6.4)	1.19, d (6.4)	1.21, d (6.0)	1.21, d (4.0)	2.16, s	2.15, m
7′	2.04, s					

**Table 2:** 13C NMR spectroscopic data (100/150 MHz) for **1**–**6** in CDCl_3_.

Position	1	2	3	4	5	6
1	163.1, C	170.4, C	162.7, C	162.6, C	162.5, C	170.2, C
3	77.4, CH	80.4, CH	78.3, CH	78.5, CH	78.3, CH	80.6, CH
4	28.0, CH_2_	32.4, CH_2_	27.4, CH_2_	33.8, CH_2_	33.7, CH_2_	32.2, CH_2_
4a	128.4, C	130.1, C	119.9, C	132.3, C	132.2, C	129.7, C
5	145.3, C	117.1, CH	147.7, CH	122.4, CH	122.4, CH	117.9, CH
6	121.2, CH	117.5, CH	106.1, C	117.5, CH	117.4, CH	120.8, CH
7	111.5, CH	147.4, C	153.1, C	153.0, C	153.0, C	143.9, C
8	155.5, C	152.5, C	142.9, C	151.6, C	151.5, C	149.1, C
8a	114.6, C	108.6, C	117.7, C	119.6, C	119.5, C	108.4, C
7-OMe		56.4, CH_3_	56.6, CH_3_	56.5, CH_3_	56.5, CH_3_	
8-OMe	56.6, CH_3_		61.8, CH_3_	61.7, CH_3_	61.7, CH_3_	
1′	34.7, CH_2_	34.8, CH_2_	34.9, CH_2_	34.8, CH_2_	34.0, CH_2_	34.1, CH_2_
2′	21.0, CH_2_	21.3, CH_2_	21.5, CH_2_	21.5, CH_2_	19.4, CH_2_	19.2, CH_2_
3′	35.7, CH_2_	38.9, CH_2_	39.1, CH_2_	39.1, CH_2_	43.2, CH_2_	43.0, CH_2_
4′	70.9, CH	67.9, CH	68.2, CH	68.1, CH	208.7, C	208.6, C
5′	20.1, CH_3_	23.7, CH_3_	23.8, CH_3_	23.8, CH_3_	30.1, CH_3_	30.1, CH_3_
6′	171.1, C					
7′	21.6, CH_3_					

**Table 3:** Antioxidant activity for compounds **2**, **6**–**11**, **17**–**19, 21** and **22**.

Compound	2	6	7	8	9	10	11	17	18	19	21	22	Trolox *^a^*
IC_50_ (mM)	28.39	0.14	0.17	0.13	0.10	0.11	0.12	0.12	0.16	0.15	20.66	0.009	0.29

*^a^* Trolox was used as a positive control.

## Data Availability

The data presented in this study are available in this article and Supplementary Material.

## Supplementary Materials

The following supporting information can be downloaded at: https://www.mdpi.com/article/10.3390/md20060361/s1, D, 2D NMR and MS data of new compounds **1**–**6**.
