# Supplementary material for: Talaromarins A–F: Six New Isocoumarins from Mangrove-Derived Fungus Talaromyces flavus TGGP35
Source: Mar Drugs. 2022 May 27;20(6):361. doi: 10.3390/md20060361 (PMC9229493; doi:10.3390/md20060361)
Supplement: Supplementary file 1 [file marinedrugs-20-00361-s001.zip › marinedrugs-1716829-supplementary.pdf]

Talaromarins A-F: Six New Isocoumarins from Mangrove-Derived Fungus *Talaromyces flavus* TGGP35

Jin Cai[1,2,†], Xiao-Chen Zhu[3,†], Wei-Nv Zeng[1,2], Bin Wang[1,2], You-Ping Luo[1,2], Jing Liu[1,2], Min-Jing Chen[1,2], Gao-Yu Li[1,2], Guo-Lei Huang[1,2], Guang-Ying Chen[1,2], Jing Xu[3,*] and Cai-Juan Zheng[1,2,*]

1 Key Laboratory of Tropical Medicinal Resource Chemistry of Ministry of Education, College of Chemistry and Chemical Engineering, Hainan Normal University, Haikou, Hainan, 571158, People's Republic of China; caijin20210207@163.com (J.C.); z17889949296@163.com (W. -N.Z.); 15180328507@163.com (B.W.); lyp188089688692022@163.com (Y.-P.L.); liujing3182799099@163.com (J.L.); cmj19151835730@163.com (M.-J. C.); qqwbma_999@163.com (G.-Y.L.); 070333@hainnu.edu.cn (G.-L.H.); chgying@163.com (G.-Y.C.)

2 Key Laboratory of Tropical Medicinal Plant Chemistry of Hainan Province, Haikou, Hainan, 571158, People's Republic of China

3 Key Laboratory of Advanced Materials of Tropical Island Resources of Ministry of Education, School of Chemical Engineering and Technology, Hainan University, Haikou 570228, China; zhuxiaochenlucky@163.com (X.-C.Z.)

* Correspondence: caijuan2002@163.com (C.-J. Z.); happyjing3@163.com (J. X.)

† These authors contributed equally to this work.

List of contents

Figure S1. ^{1}H NMR spectrum of **1** in $CDCl_3$
Figure S2. ^{13}C NMR spectrum of **1** in $CDCl_3$
Figure S3. 135-DEPT spectrum of **1** in $CDCl_3$
Figure S4. HMQC spectrum of **1** in $CDCl_3$
Figure S5. ^{1}H-^{1}H COSY spectrum of **1** in $CDCl_3$
Figure S6. HMBC spectrum of **1** in $CDCl_3$
Figure S7. HR-ESI-MS spectrum of **1**

Figure S8. ^{1}H NMR spectrum of **2** in $CDCl_3$
Figure S9. ^{13}C NMR spectrum of **2** in $CDCl_3$
Figure S10. 135-DEPT spectrum of **2** in $CDCl_3$
Figure S11. HMQC spectrum of **2** in $CDCl_3$
Figure S12. ^{1}H-^{1}H COSY spectrum of **2** in $CDCl_3$
Figure S13. HMBC spectrum of **2** in $CDCl_3$
Figure S14. HR-ESI-MS spectrum of **2**

Figure S15. ^{1}H NMR spectrum of **3** in $CDCl_3$
Figure S16. ^{13}C NMR spectrum of **3** in $CDCl_3$
Figure S17. 135-DEPT spectrum of **3** in $CDCl_3$
Figure S18. HMQC spectrum of **3** in $CDCl_3$
Figure S19. ^{1}H-^{1}H COSY spectrum of **3** in $CDCl_3$
Figure S20. HMBC spectrum of **3** in $CDCl_3$
Figure S21. HR-ESI-MS spectrum of **3**

Figure S22. ^{1}H NMR spectrum of **4** in $CDCl_3$
Figure S23. ^{13}C NMR spectrum of **4** in $CDCl_3$
Figure S24. 135-DEPT spectrum of **4** in $CDCl_3$
Figure S25. HMQC spectrum of **4** in $CDCl_3$
Figure S26. ^{1}H-^{1}H COSY spectrum of **4** in $CDCl_3$
Figure S27. HMBC spectrum of **4** in $CDCl_3$
Figure S28. HR-ESI-MS spectrum of **4**

Figure S29. ^{1}H NMR spectrum of **5** in $CDCl_3$
Figure S30. ^{13}C NMR spectrum of **5** in $CDCl_3$
Figure S31. 135-DEPT spectrum of **5** in $CDCl_3$
Figure S32. HMQC spectrum of **5** in $CDCl_3$
Figure S33. ^{1}H-^{1}H COSY spectrum of **5** in $CDCl_3$
Figure S34. HMBC spectrum of **5** in $CDCl_3$
Figure S35. HR-ESI-MS spectrum of **5**

Figure S36. ^{1}H NMR spectrum of **6** in $CDCl_3$
Figure S37. ^{13}C NMR spectrum of **6** in $CDCl_3$
Figure S38. 135-DEPT spectrum of **6** in $CDCl_3$
Figure S39. HMQC spectrum of **6** in $CDCl_3$
Figure S40. ^{1}H-^{1}H COSY spectrum of **6** in $CDCl_3$
Figure S41. HMBC spectrum of **6** in $CDCl_3$
Figure S42. HR-ESI-MS spectrum of **6**

Figure S**43**. ^{1}H NMR ($CDCl_3$, 400 MHz) of Hydrolysate of **1**a
Figure S**44**. ^{13}C NMR ($CDCl_3$, 400 MHz) of Hydrolysate of **1**a
Figure S**45**. ESI-MS spectrum of **1a**
Figure S**46**. ^{1}H NMR ($CDCl_3$, 600 MHz) of *S*-MTPA ester of **1**a
Figure S**47**. ESI-MS spectrum of **1**a
Figure S**48**. ^{1}H NMR ($CDCl_3$, 600 MHz) of *R*-MTPA ester of **1**a
Figure S**49**. ESI-MS spectrum of **1**a

Figure S**50**. ^{1}H NMR ($CDCl_3$, 600 MHz) of *S*-MTPA ester of **2**
Figure S**51**. ESI-MS spectrum of **2**
Figure S**52**. ^{1}H NMR ($CDCl_3$, 600 MHz) of *R*-MTPA ester of **2**
Figure S**53**. ESI-MS spectrum of **2**

Figure S**54**. ^{1}H NMR ($CDCl_3$, 400 MHz) of *S*-MTPA ester of **3**
Figure S**55**. ESI-MS spectrum of **3**
Figure S**56**. ^{1}H NMR ($CDCl_3$, 400 MHz) of *R*-MTPA ester of **3**
Figure S**57**. ESI-MS spectrum of **3**

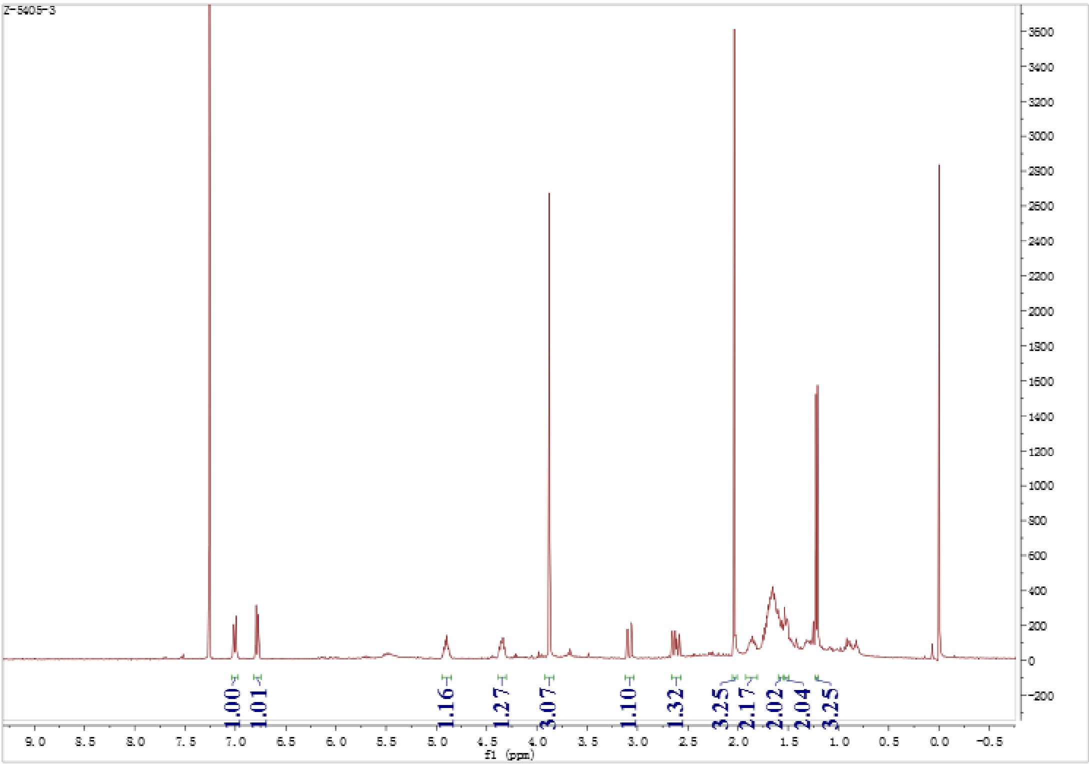

Figure S1. ^{1}H NMR spectrum of **1** in $CDCl_3$

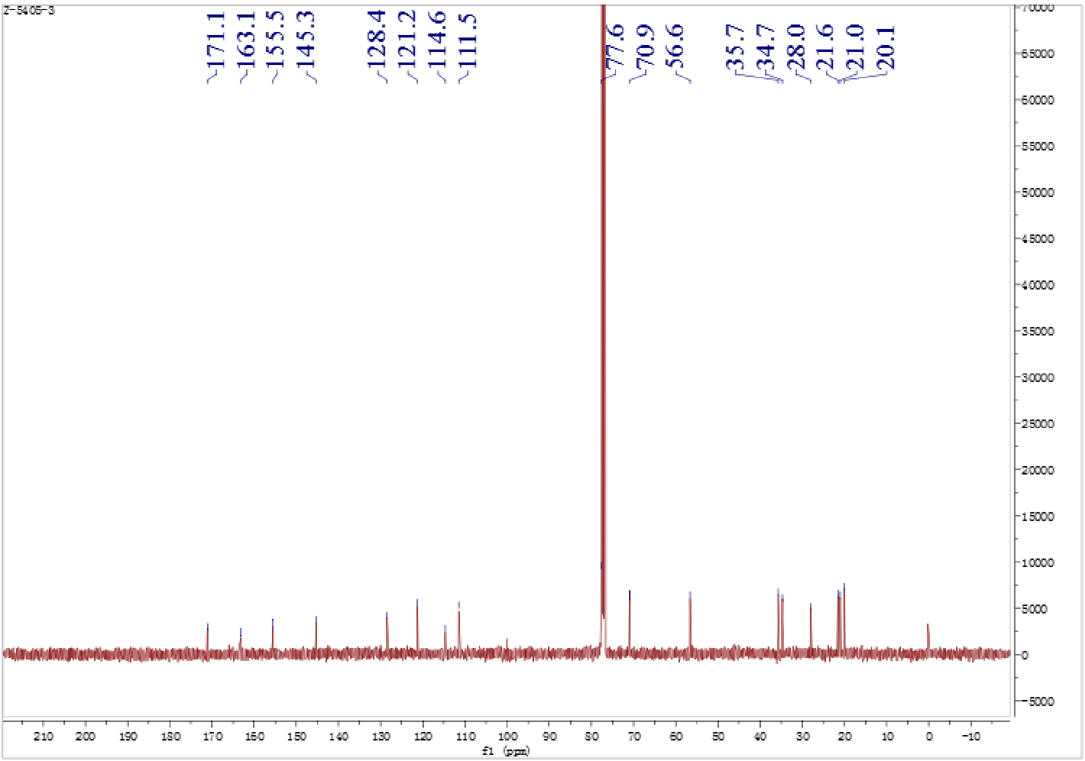

Figure S2. ^{13}C NMR spectrum of **1** in $CDCl_3$

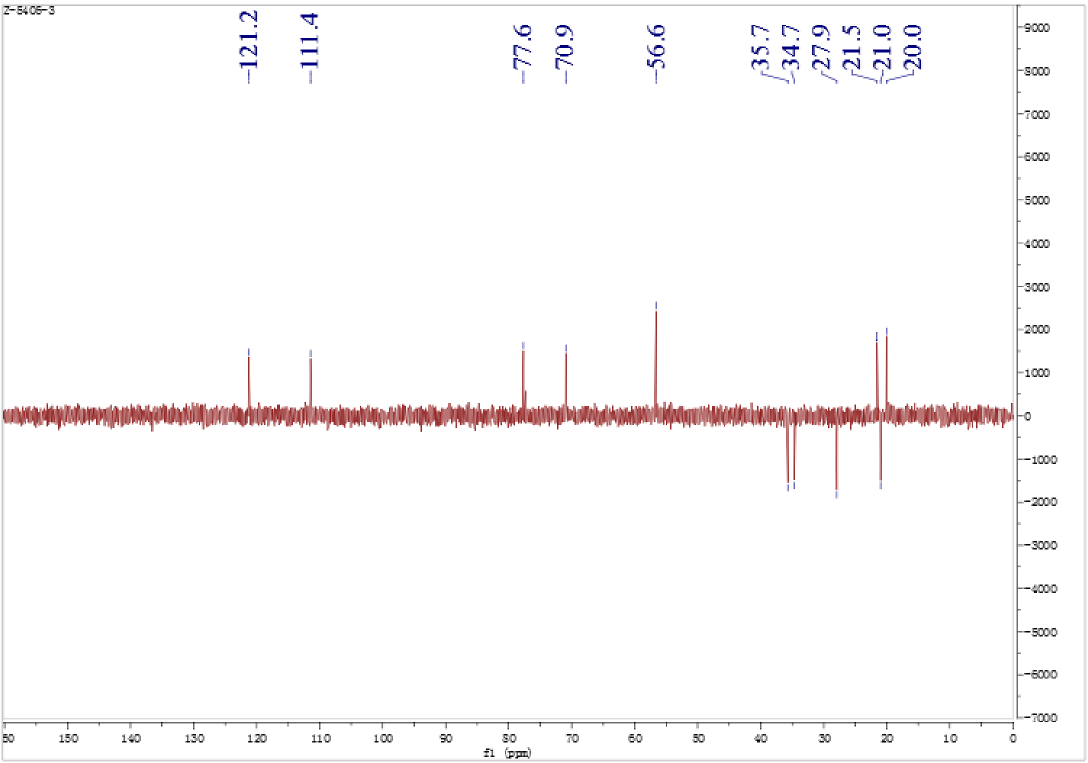

Figure S3. 135-DEPT spectrum of **1** in $CDCl_3$

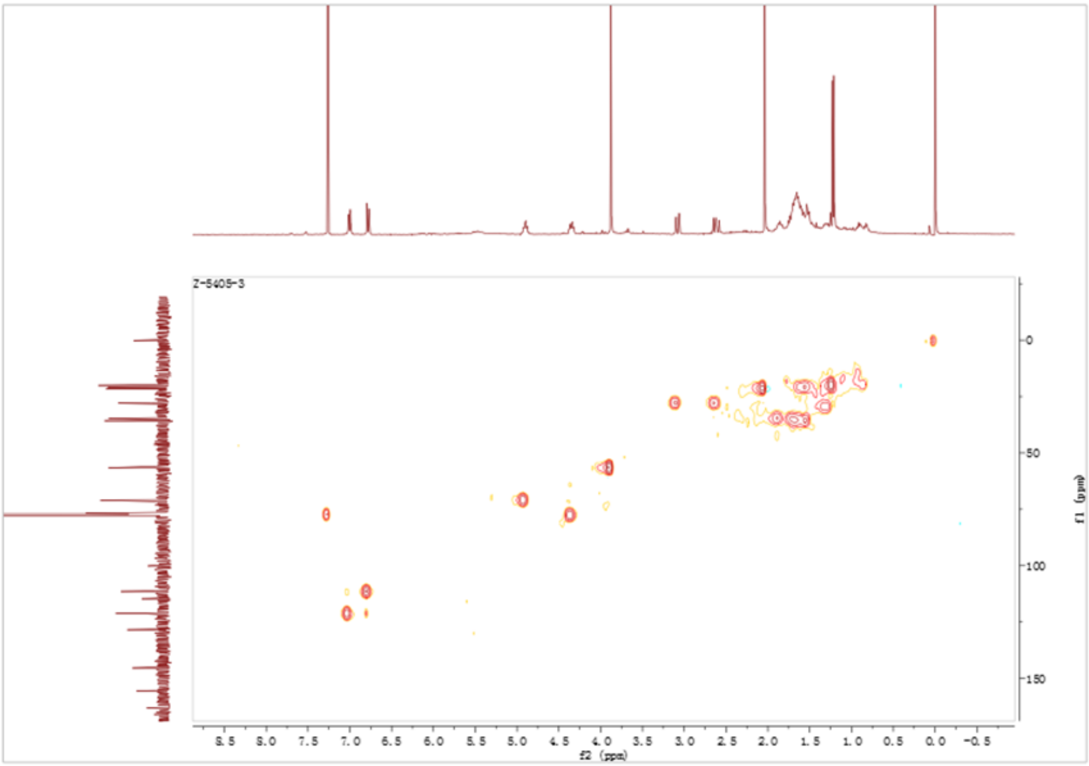

Figure S4. HMQC spectrum of **1** in $CDCl_3$

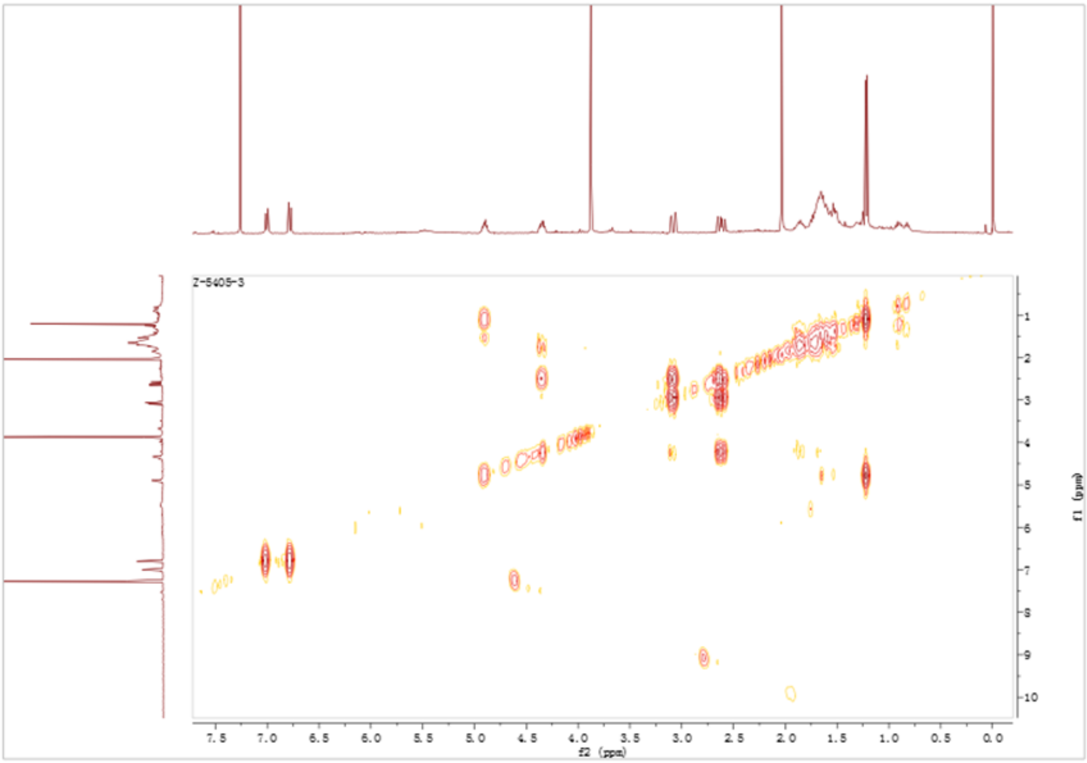

Figure S5. ^{1}H-^{1}H COSY spectrum of **1** in $CDCl_3$

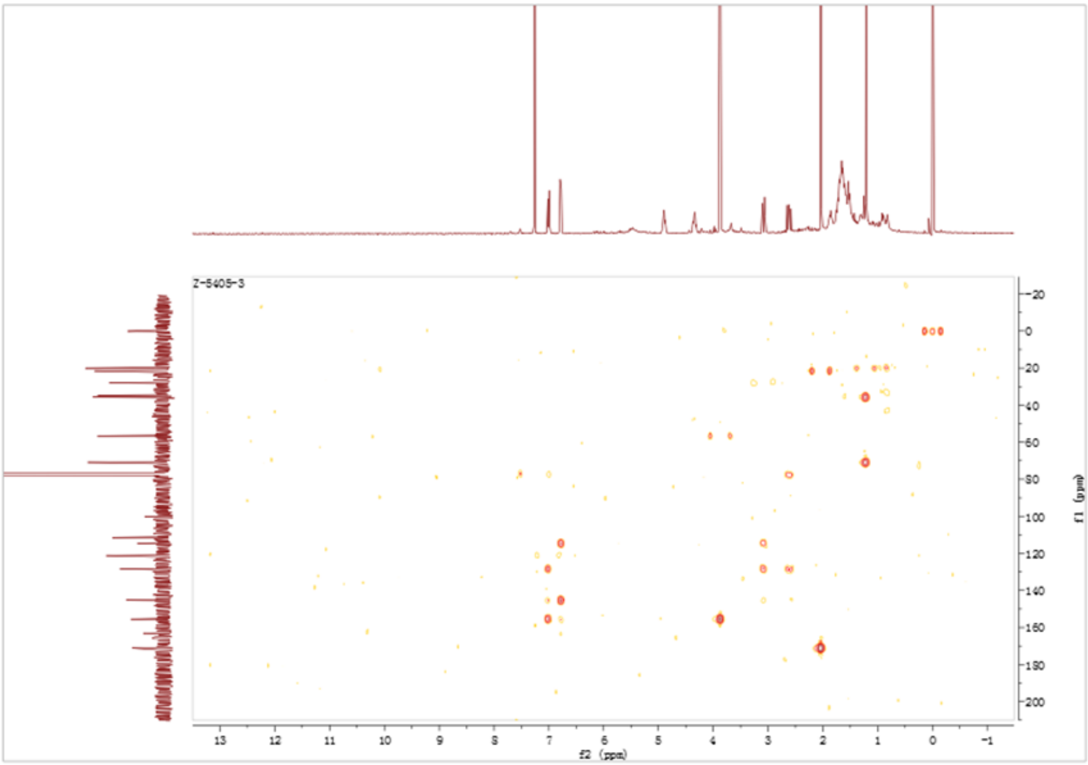

Figure S6. HMBC spectrum of **1** in $CDCl_3$

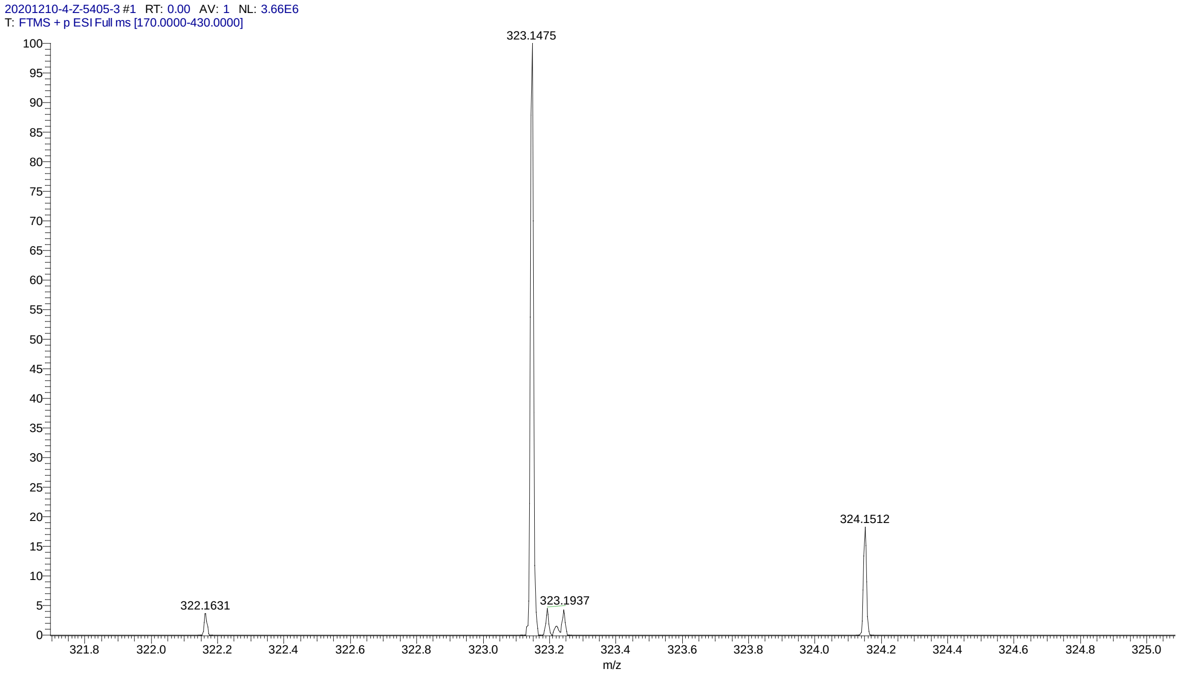

Figure S7. HR-ESI-MS spectrum of **1**

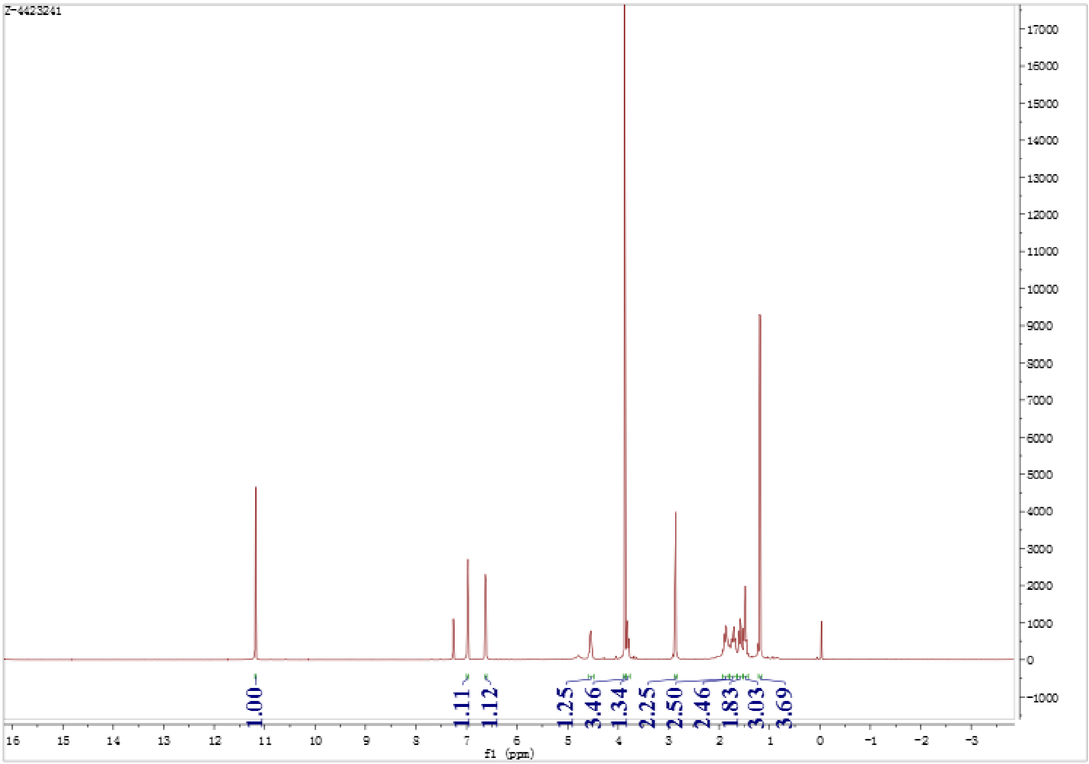

Figure S8. ^{1}H NMR spectrum of **2** in $CDCl_3$

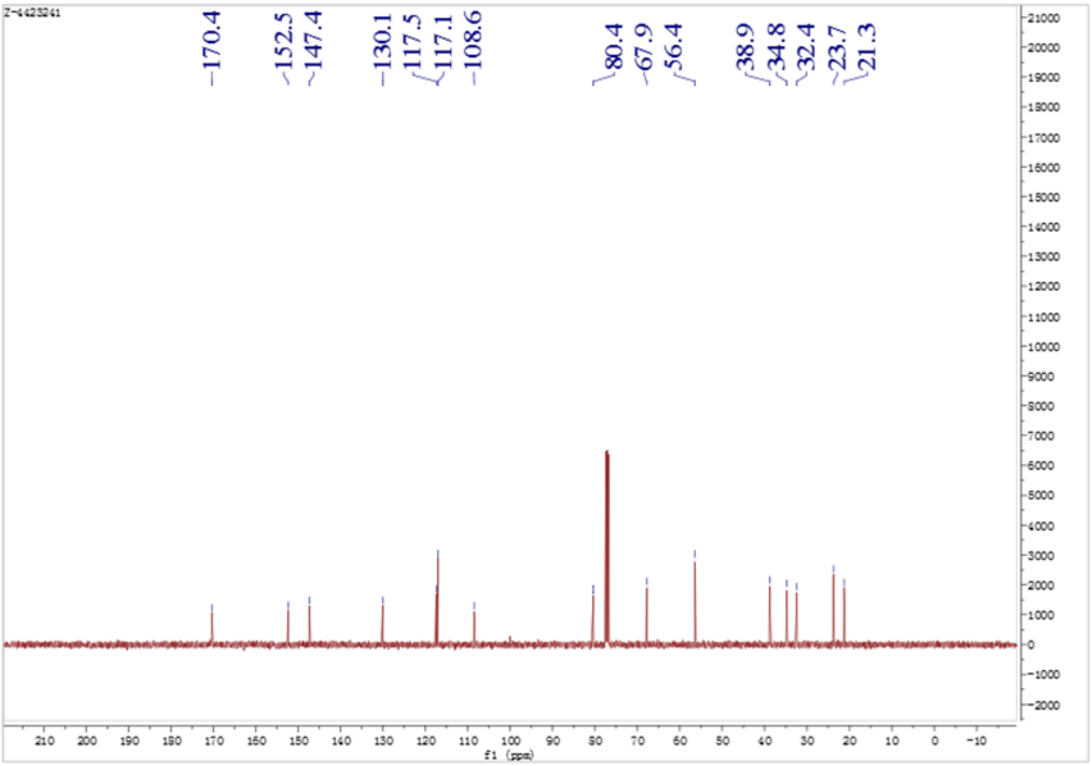

Figure S9. ^{13}C NMR spectrum of **2** in $CDCl_3$

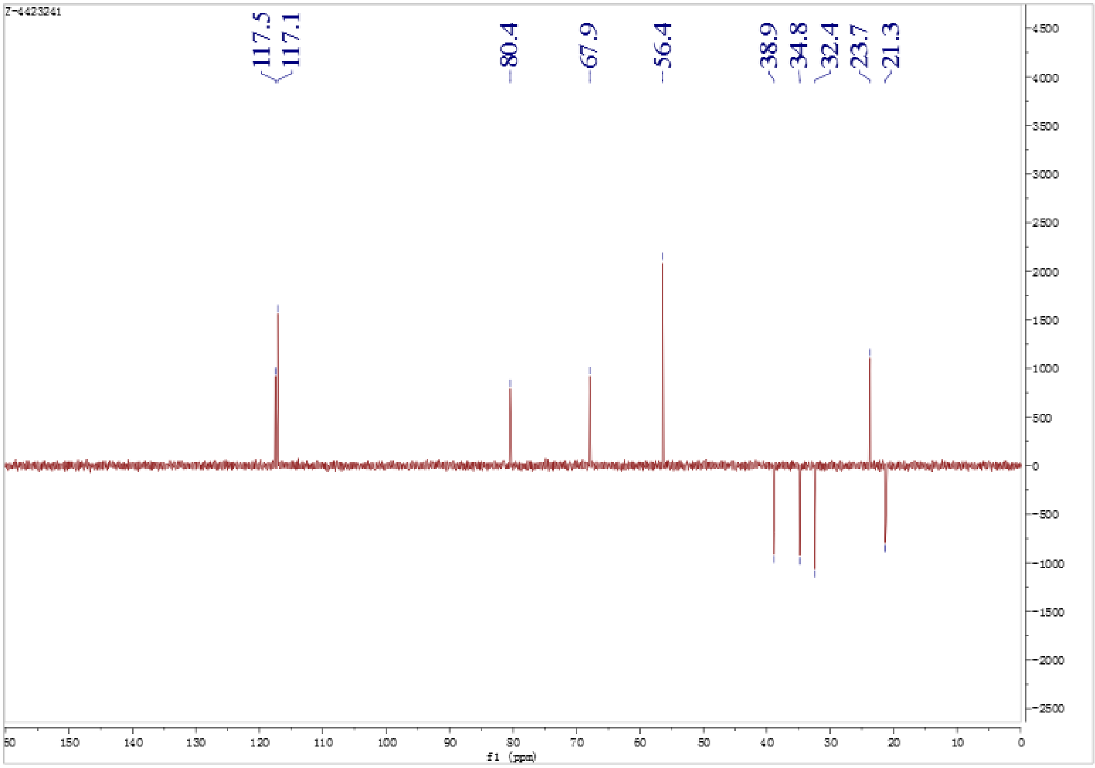

Figure S10. 135-DEPT spectrum of **2** in $CDCl_3$

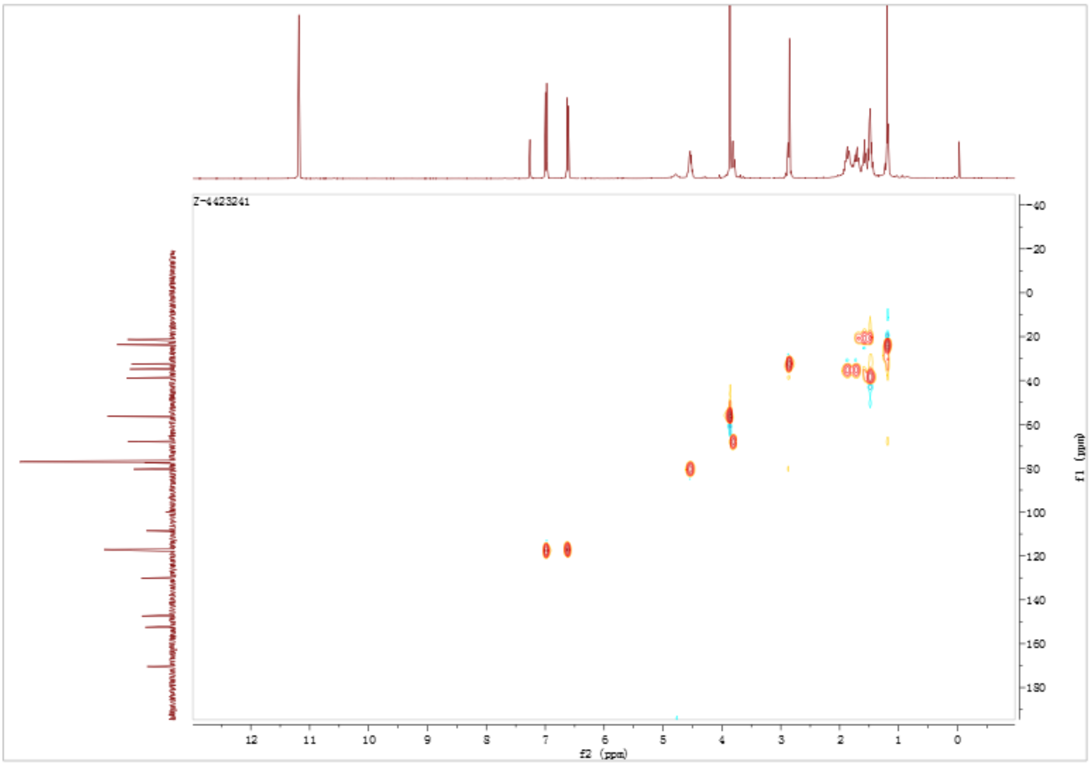

Figure S11. HMQC spectrum of **2** in $CDCl_3$

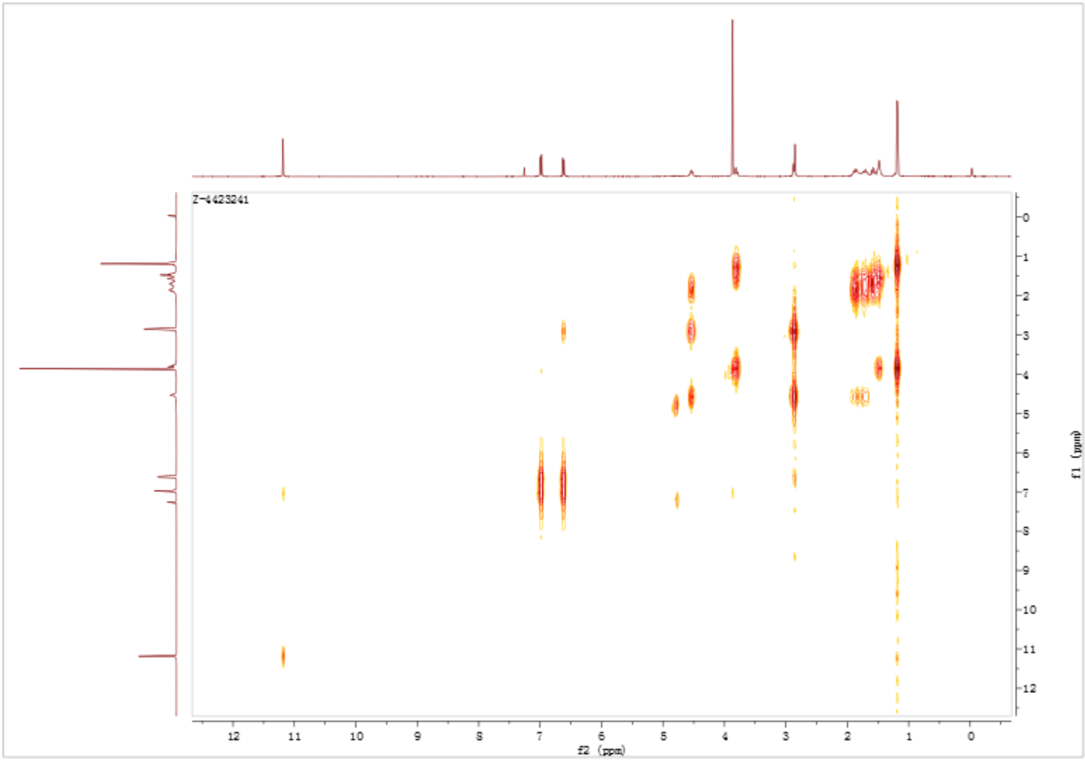

Figure S12. ^{1}H-^{1}H COSY spectrum of **2** in $CDCl_3$

`

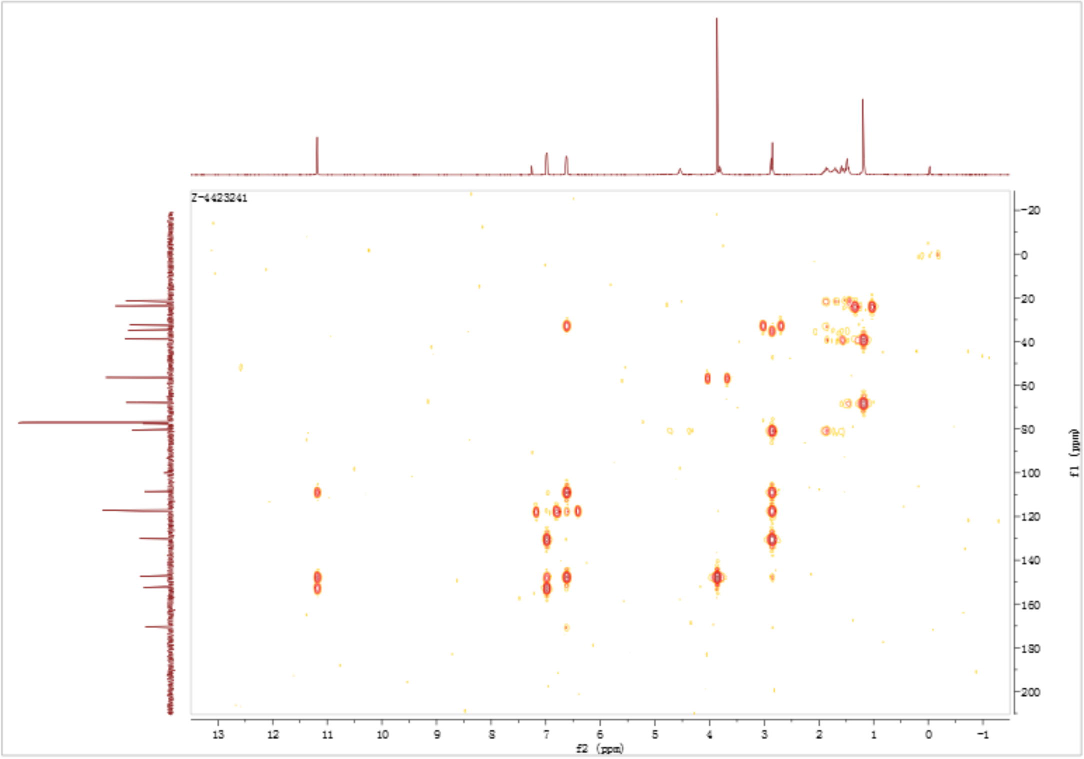

Figure S13. HMBC spectrum of **2** in $CDCl_3$

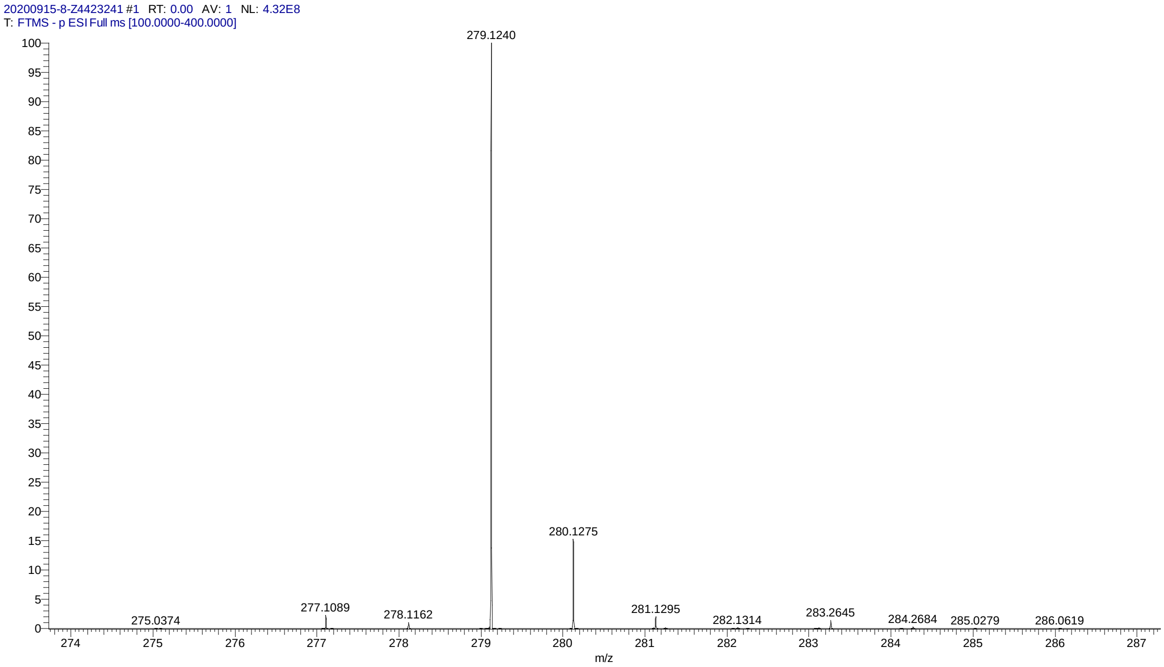

Figure S14. HR-ESI-MS spectrum of **2**

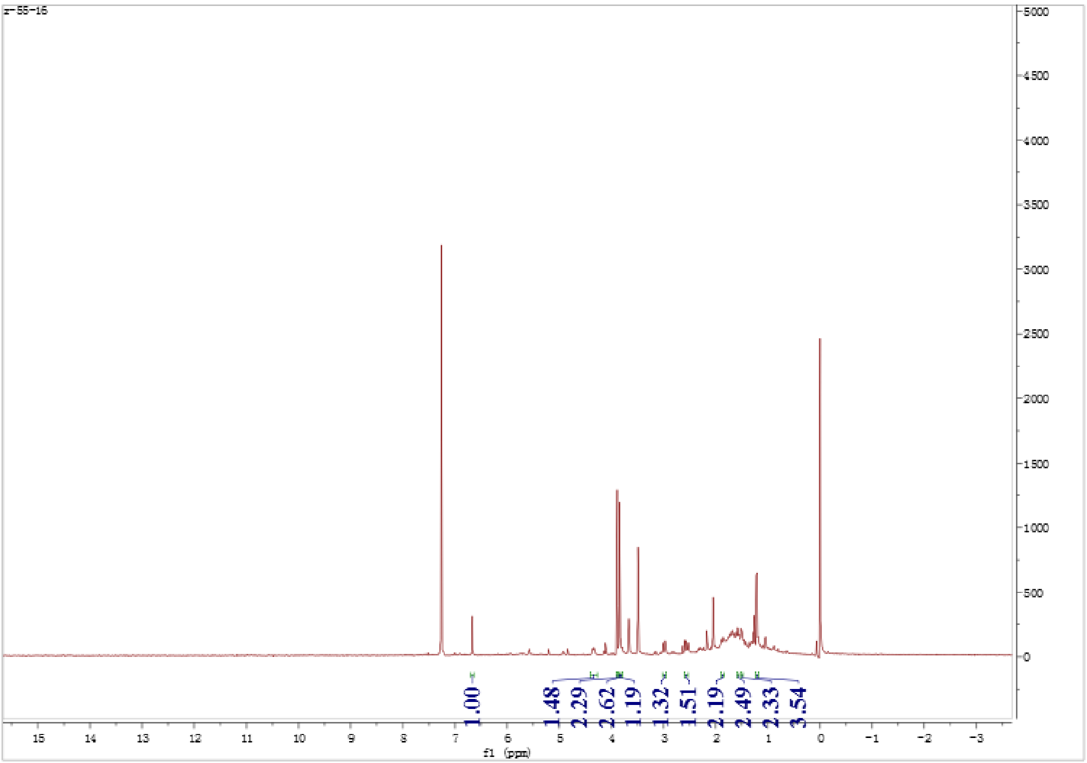

Figure S15. ^{1}H NMR spectrum of **3** in $CDCl_3$

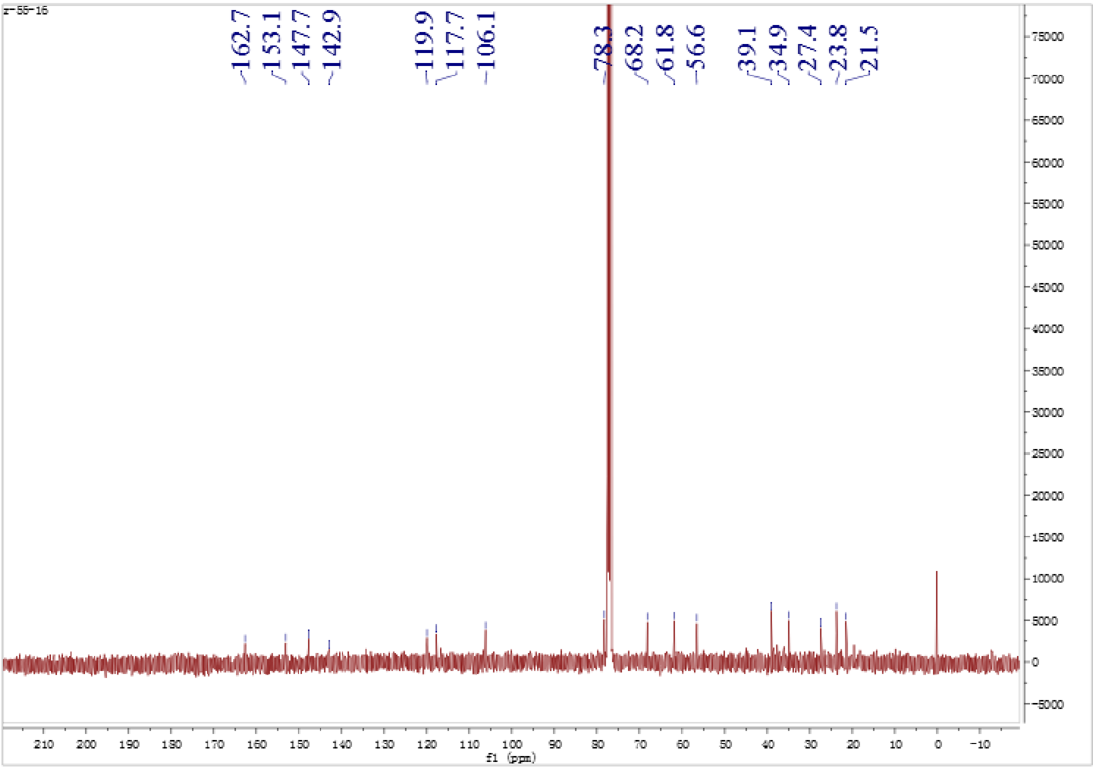

Figure S16. ^{13}C NMR spectrum of **3** in $CDCl_3$

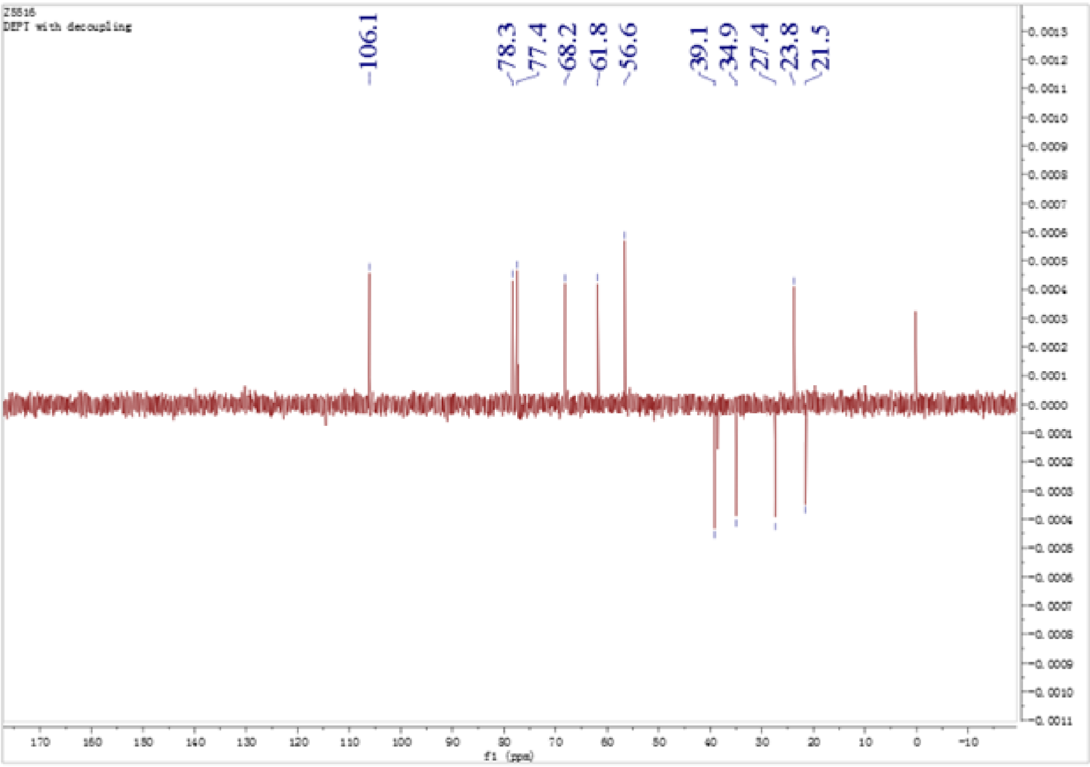

Figure S17. 135-DEPT spectrum of **2** in $CDCl_3$

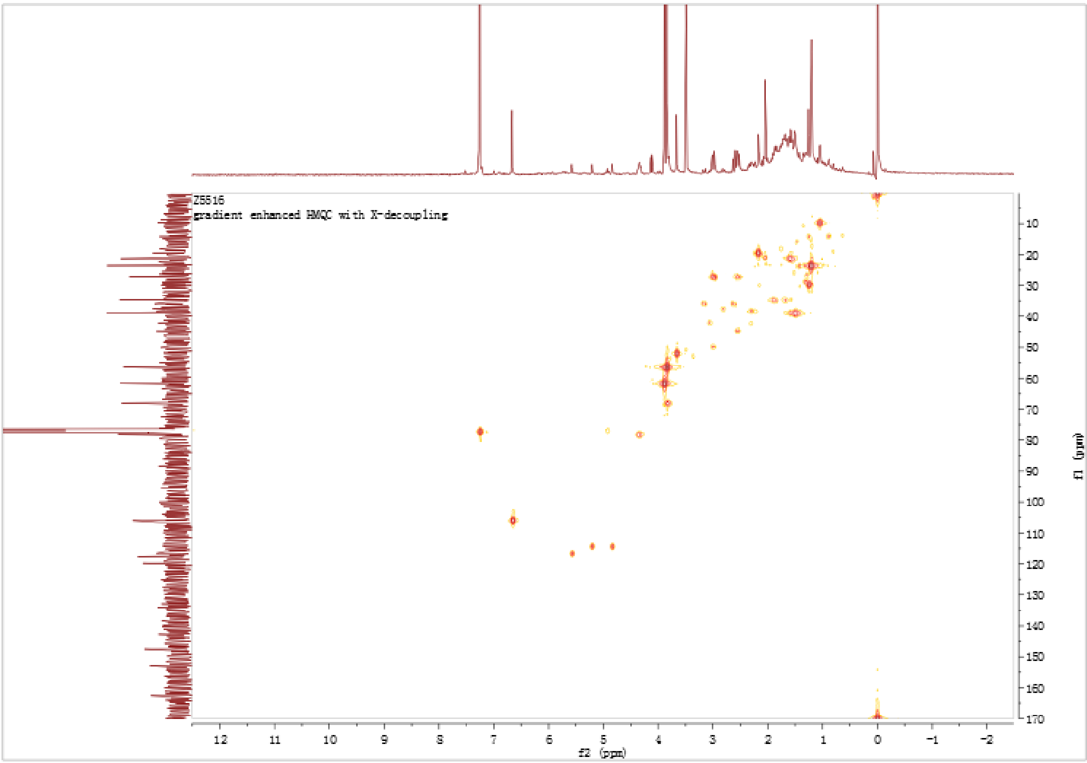

Figure S18. HMQC spectrum of **3** in $CDCl_3$

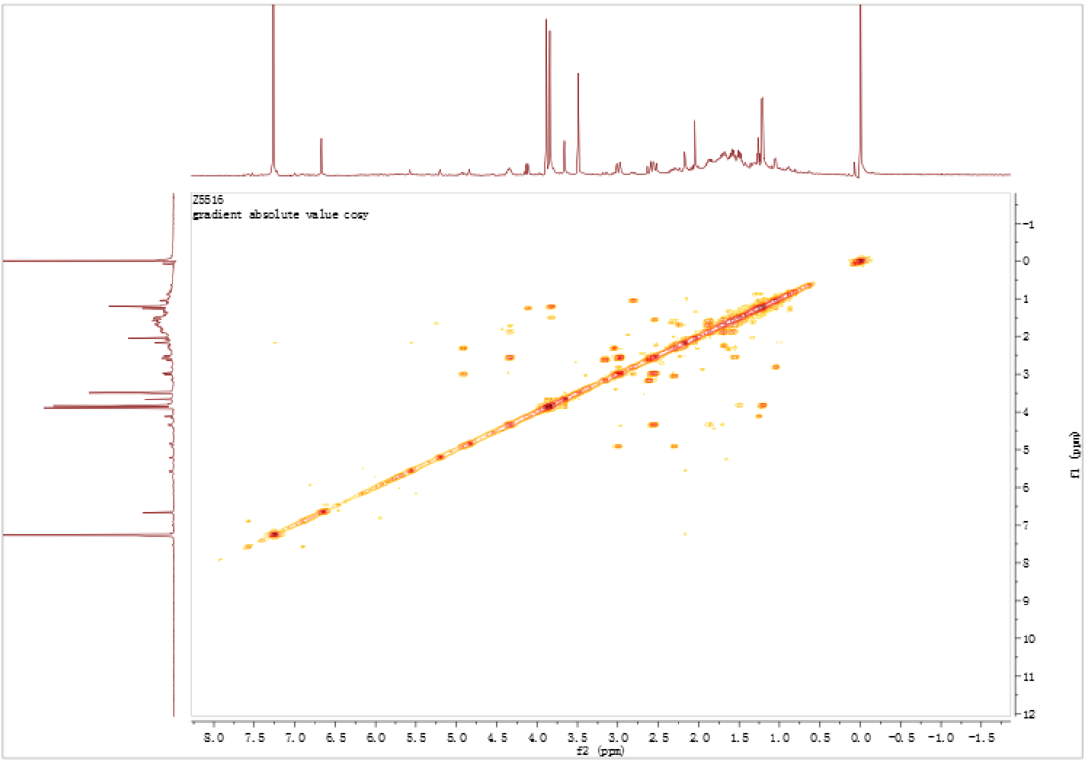

Figure S19. ^{1}H-^{1}H COSY spectrum of **2** in $CDCl_3$

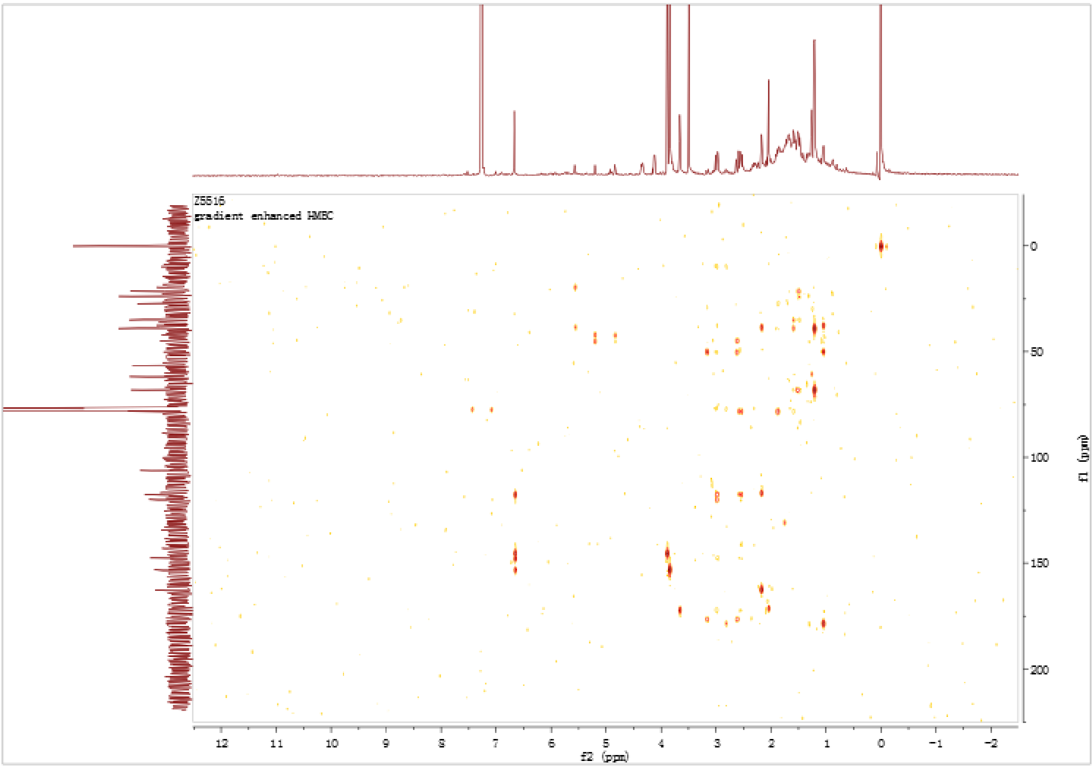

Figure S20. HMBC spectrum of **3** in $CDCl_3$

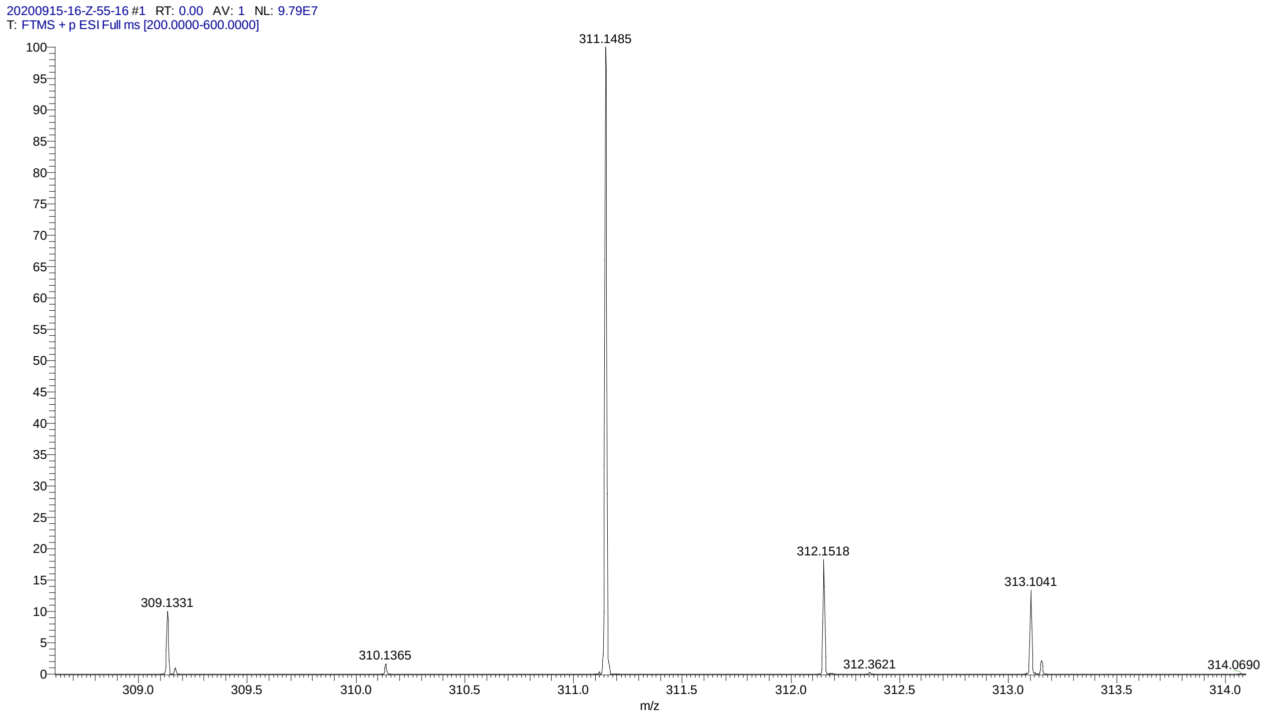

Figure S21. HR-ESI-MS spectrum of **3**

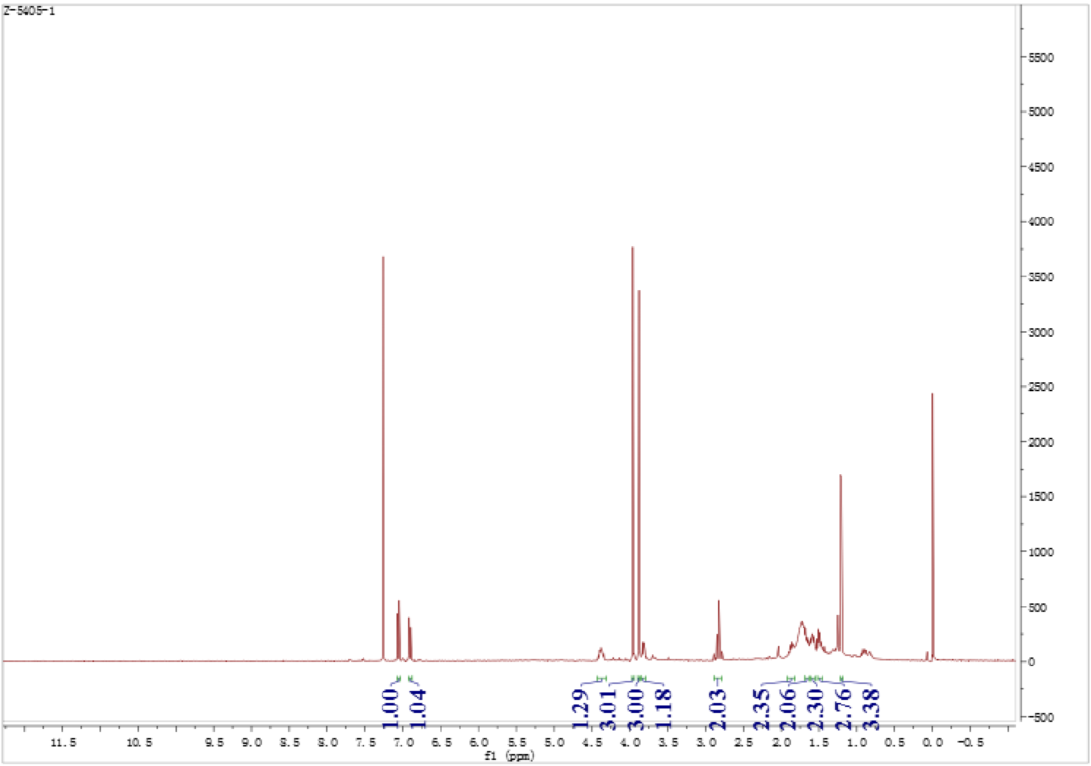

Figure S22. ^{1}H NMR spectrum of **4** in $CDCl_3$

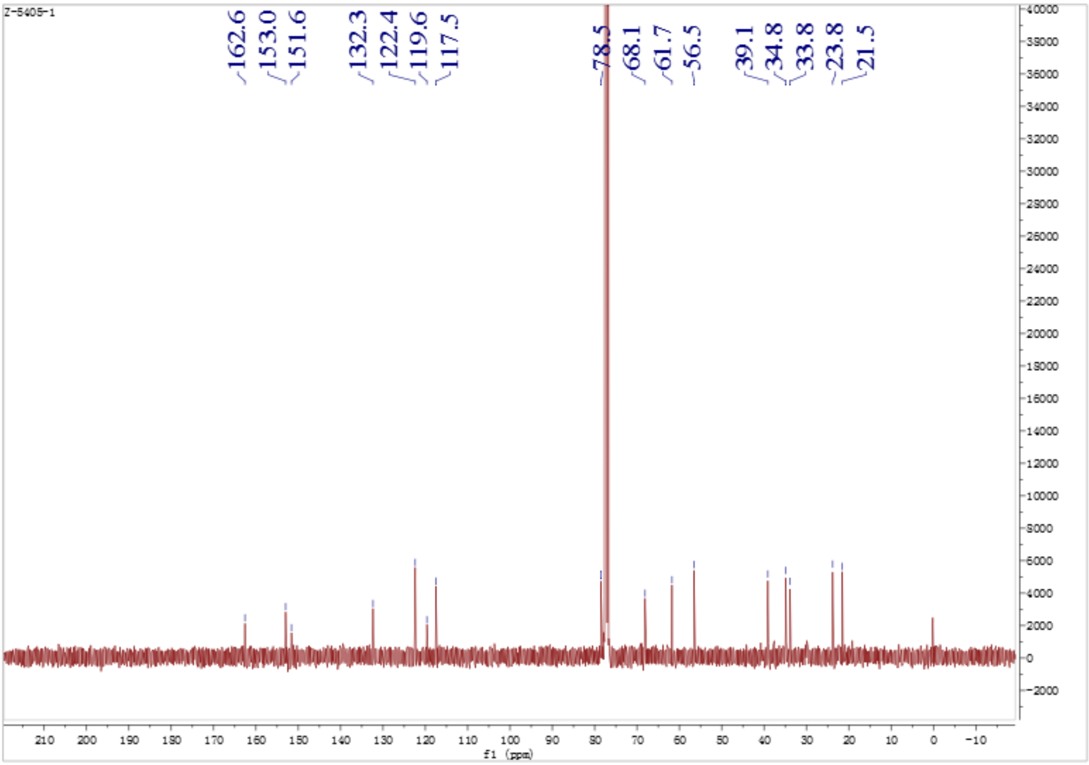

Figure S23. ^{13}C NMR spectrum of **4** in $CDCl_3$

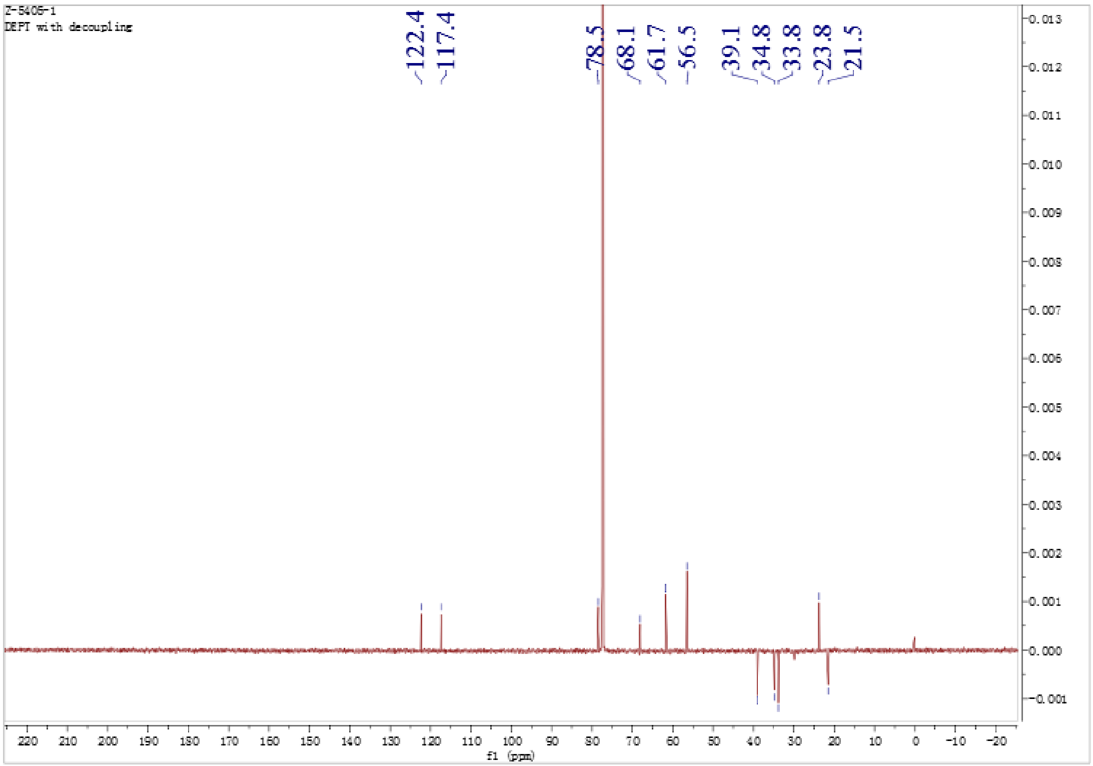

Figure S24. 135-DEPT spectrum of **4** in $CDCl_3$

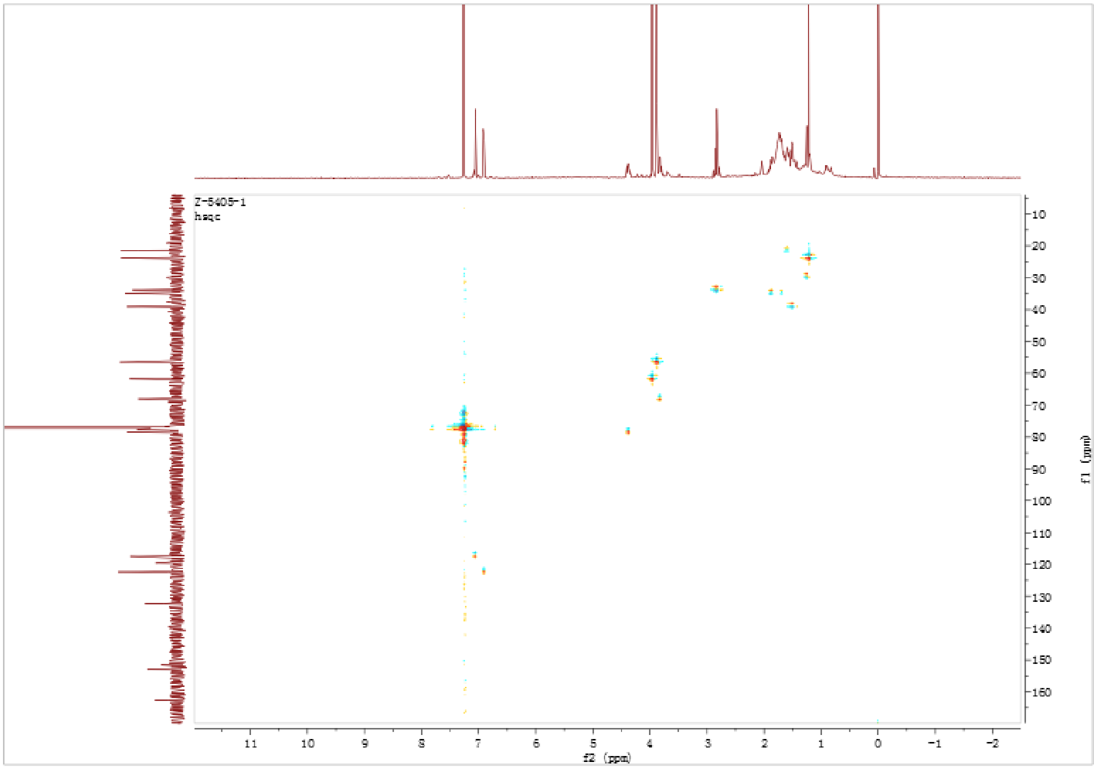

Figure S25. HMQC spectrum of **4** in $CDCl_3$

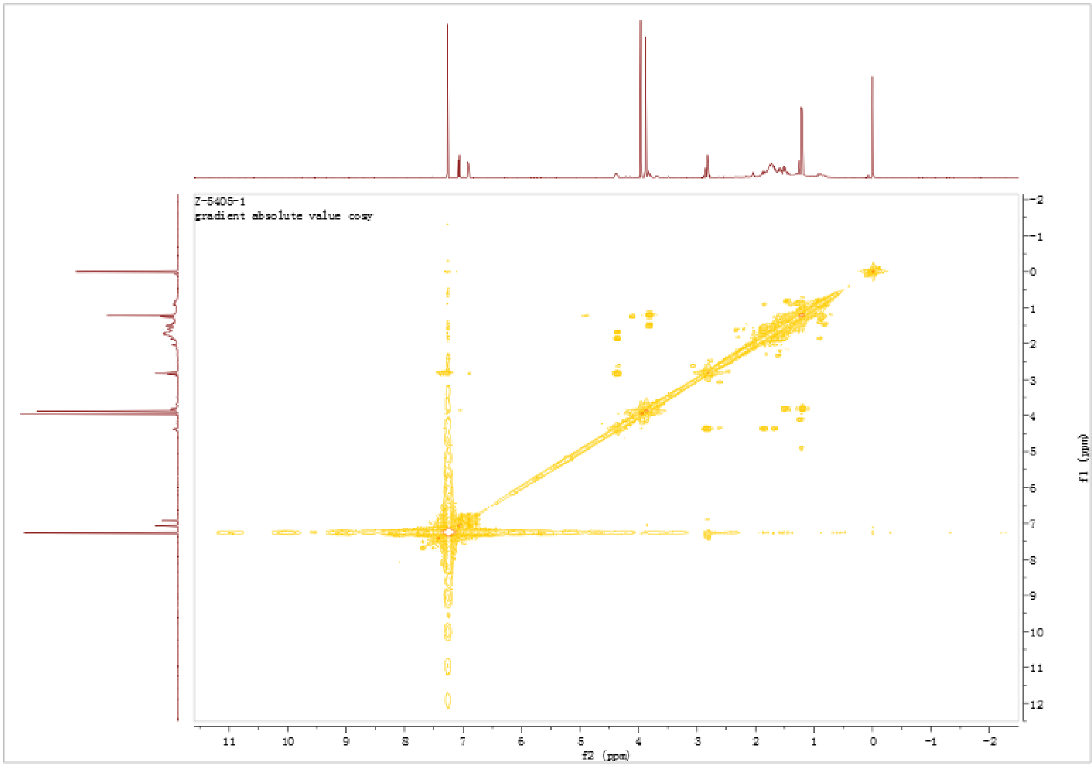

Figure S26. ^{1}H-^{1}H COSY spectrum of **4** in $CDCl_3$

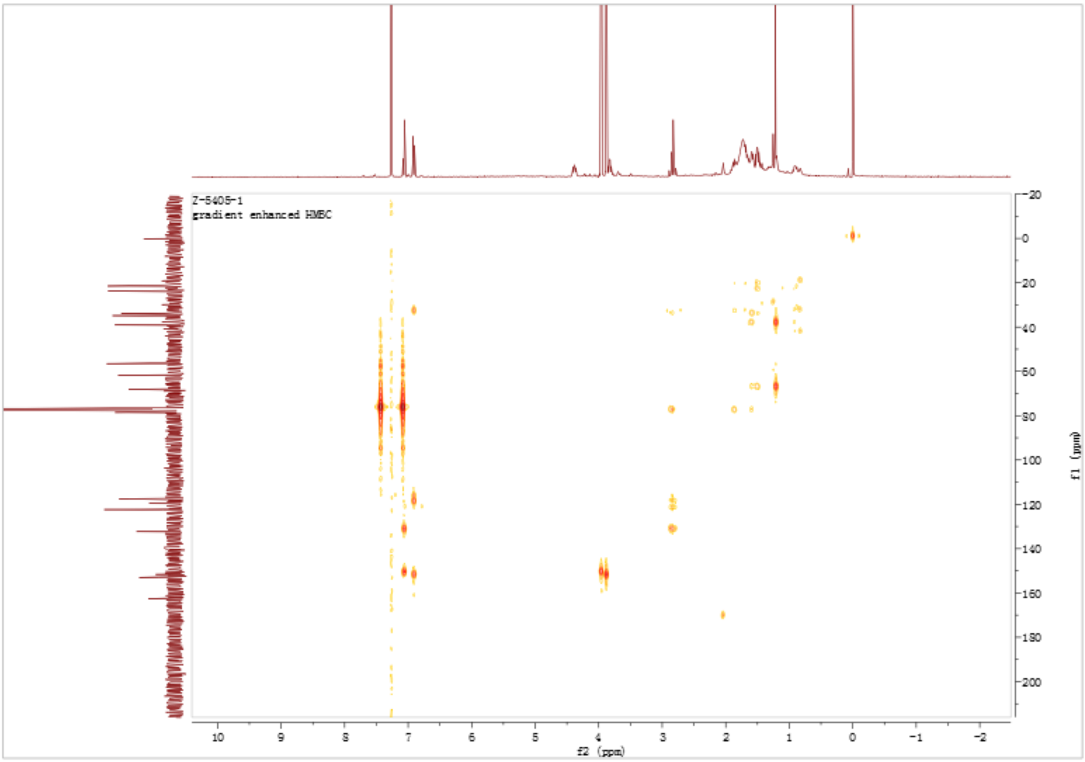

Figure S27. HMBC spectrum of **4** in $CDCl_3$

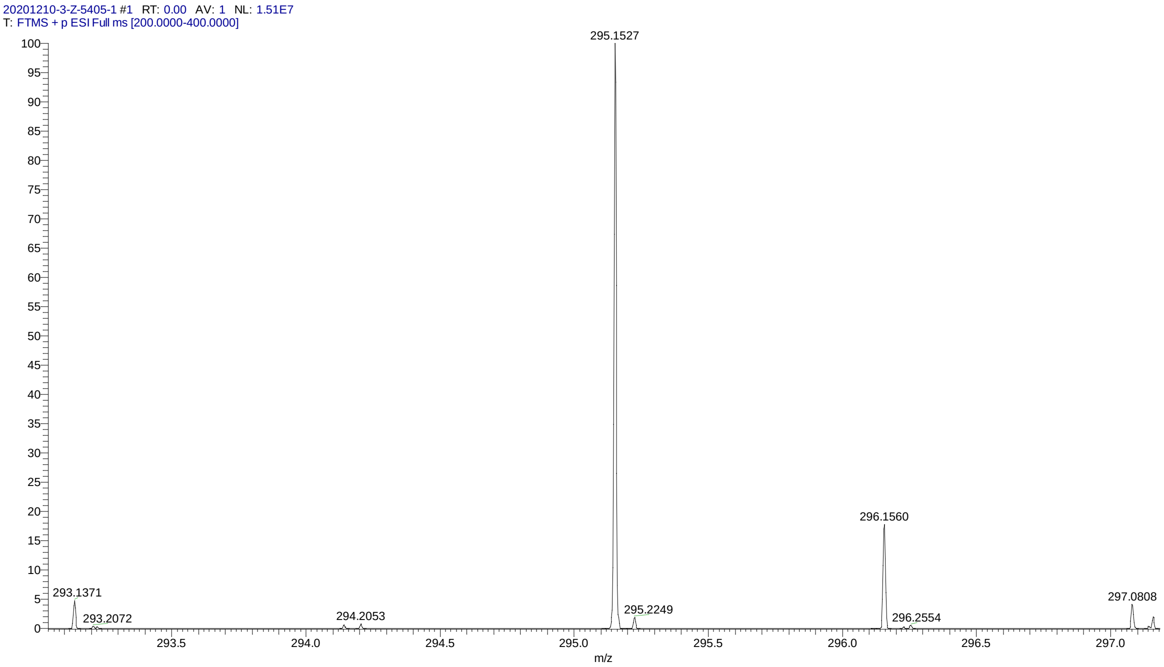

Figure S28. HR-ESI-MS spectrum of **4**

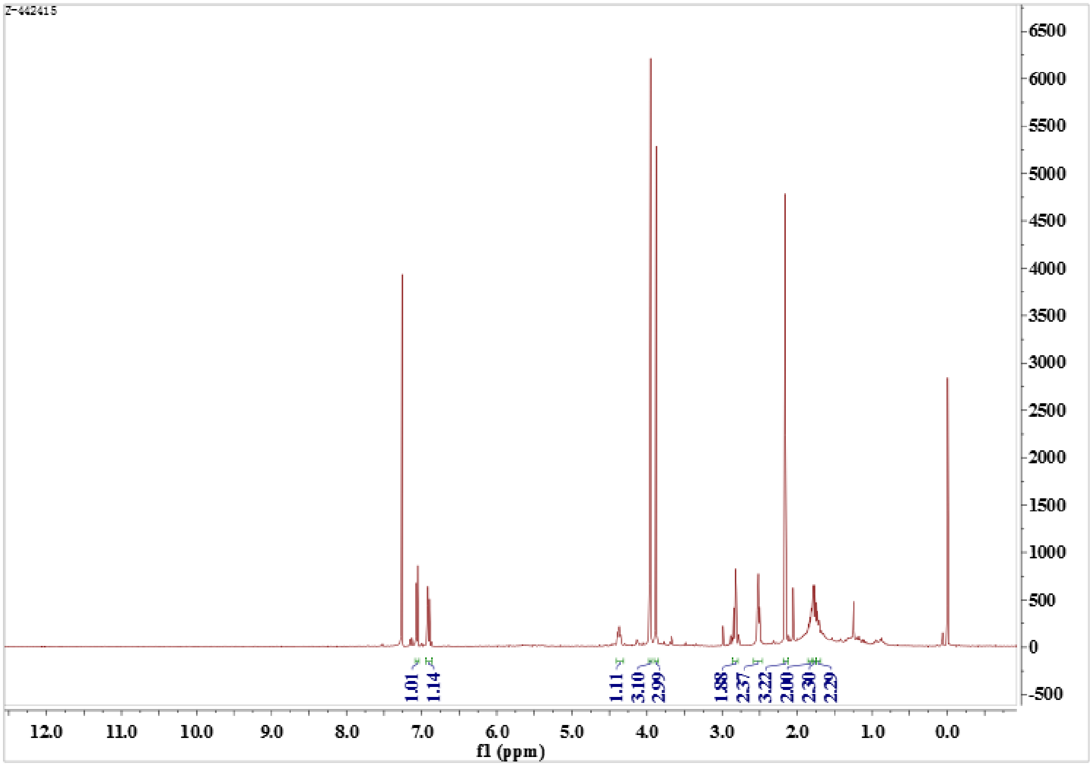

Figure S29. ^{1}H NMR spectrum of **5** in $CDCl_3$

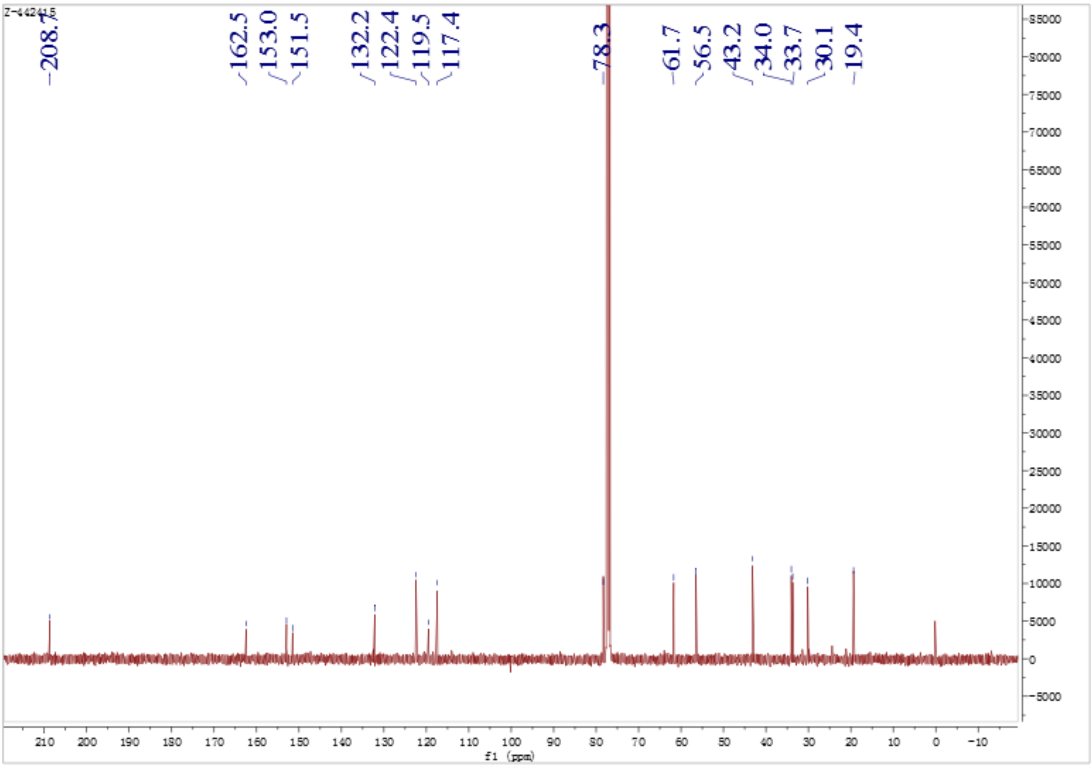

Figure S30. ^{13}C NMR spectrum of **5** in $CDCl_3$

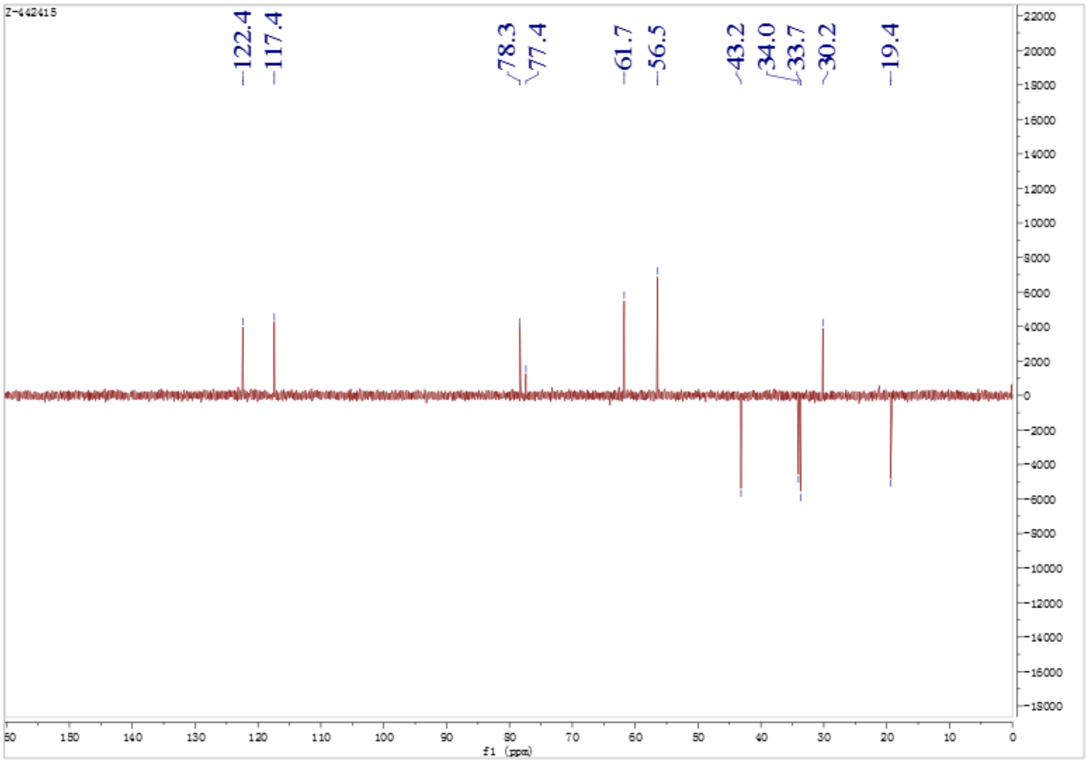

Figure S31. 135-DEPT spectrum of **5** in $CDCl_3$

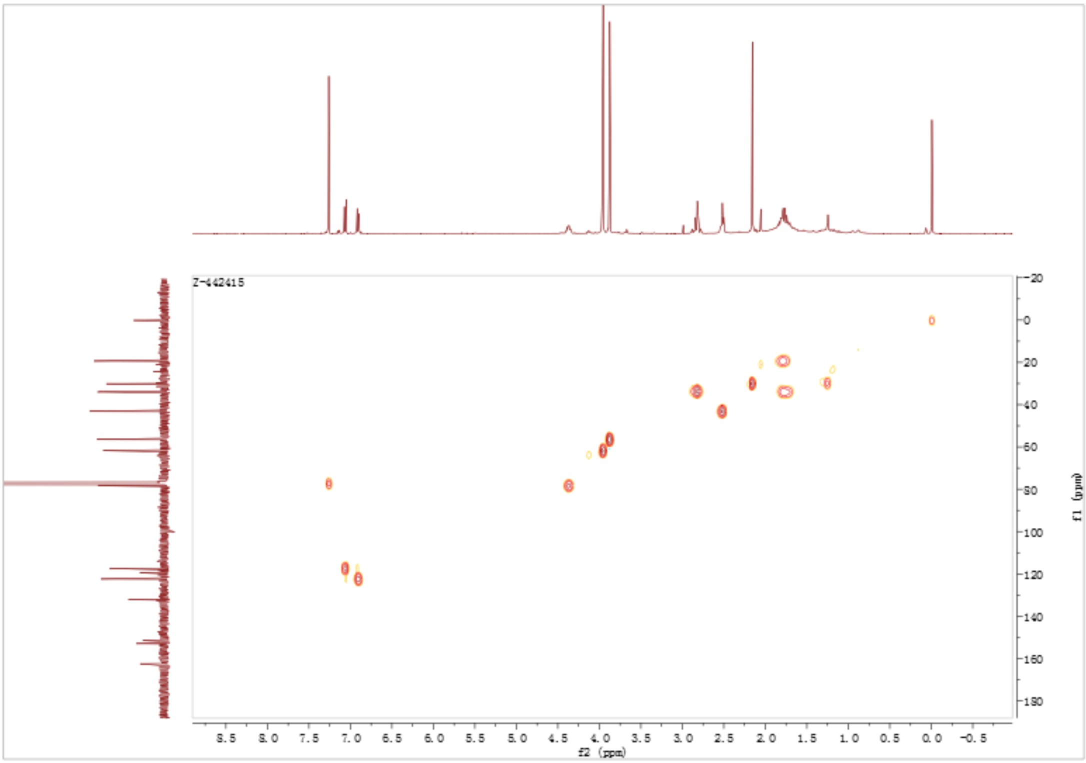

Figure S32. HMQC spectrum of **5** in $CDCl_3$

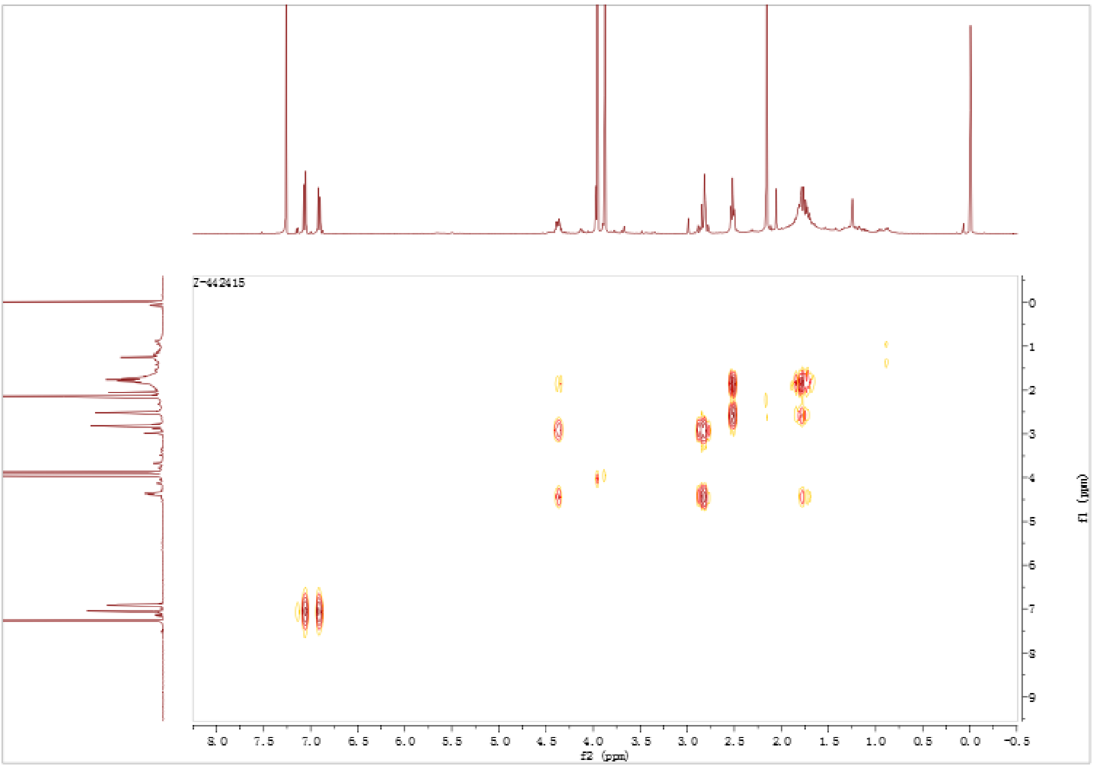

Figure S33. ^{1}H-^{1}H COSY spectrum of **5** in $CDCl_3$

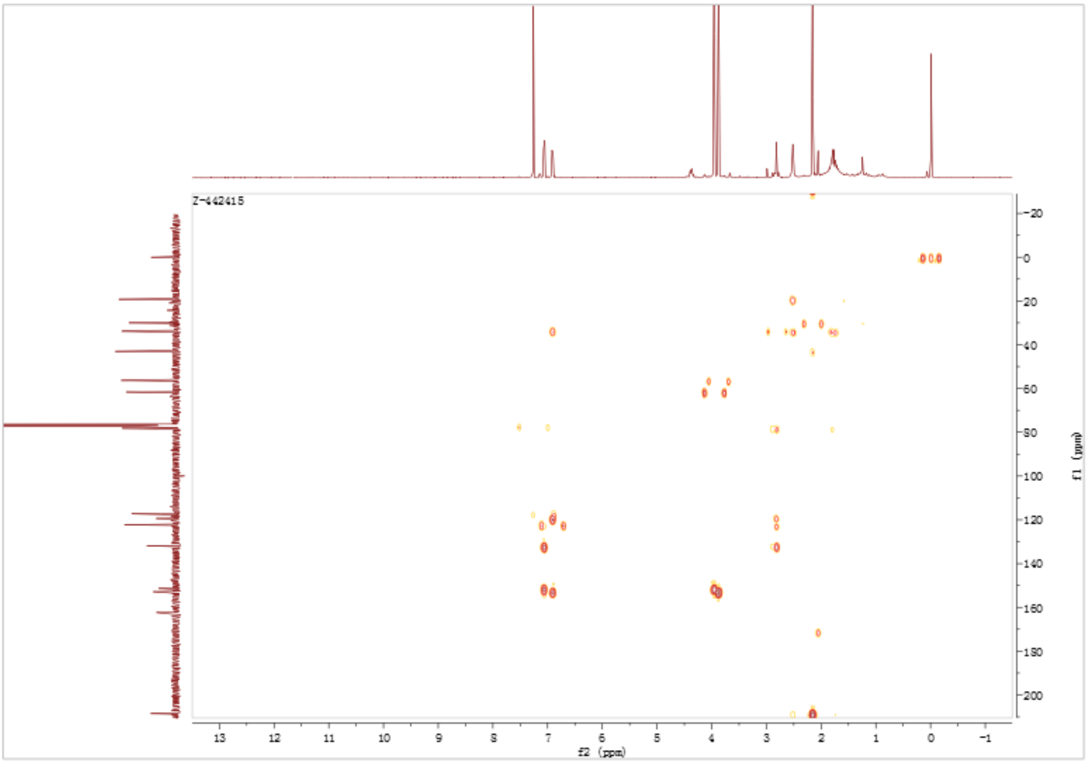

Figure S34. HMBC spectrum of **5** in $CDCl_3$

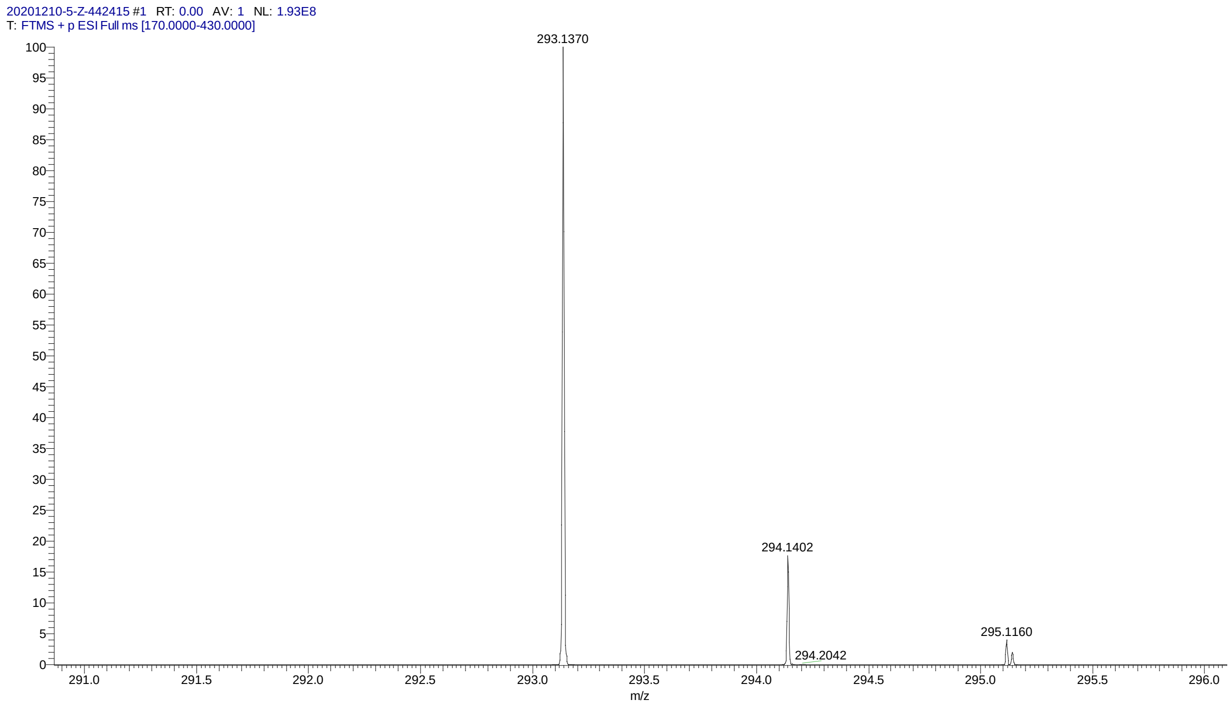

Figure S35. HR-ESI-MS spectrum of **5**

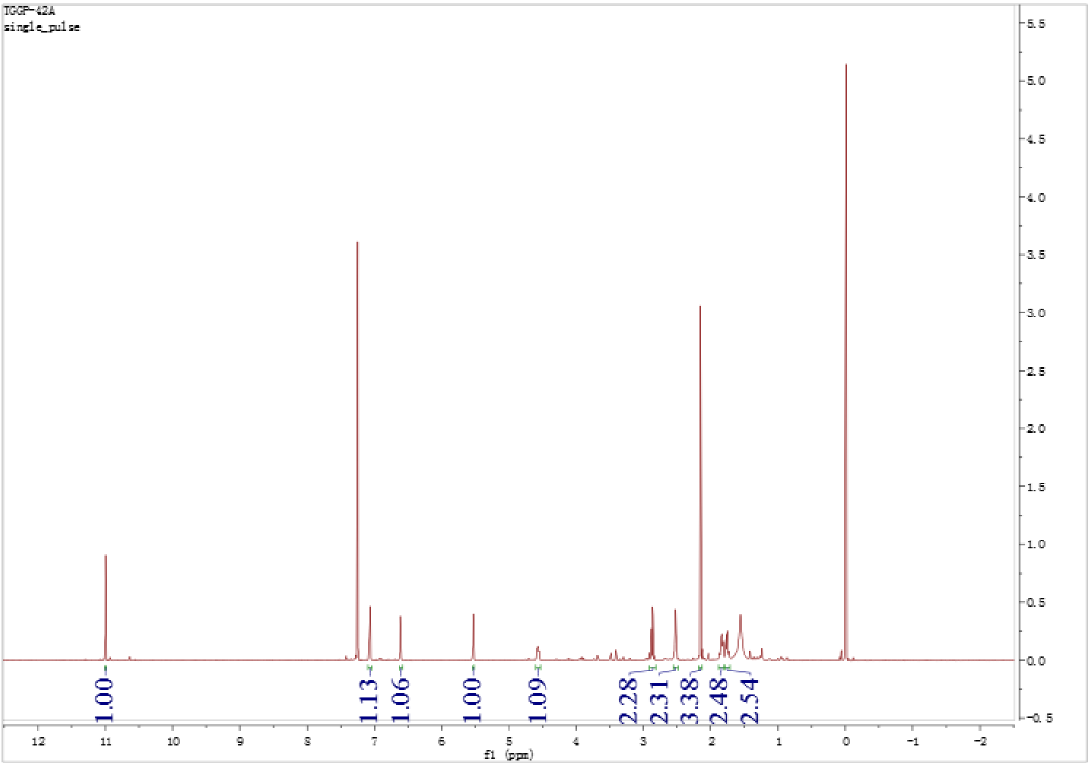

Figure S36. ^{1}H NMR spectrum of **6** in $CDCl_3$

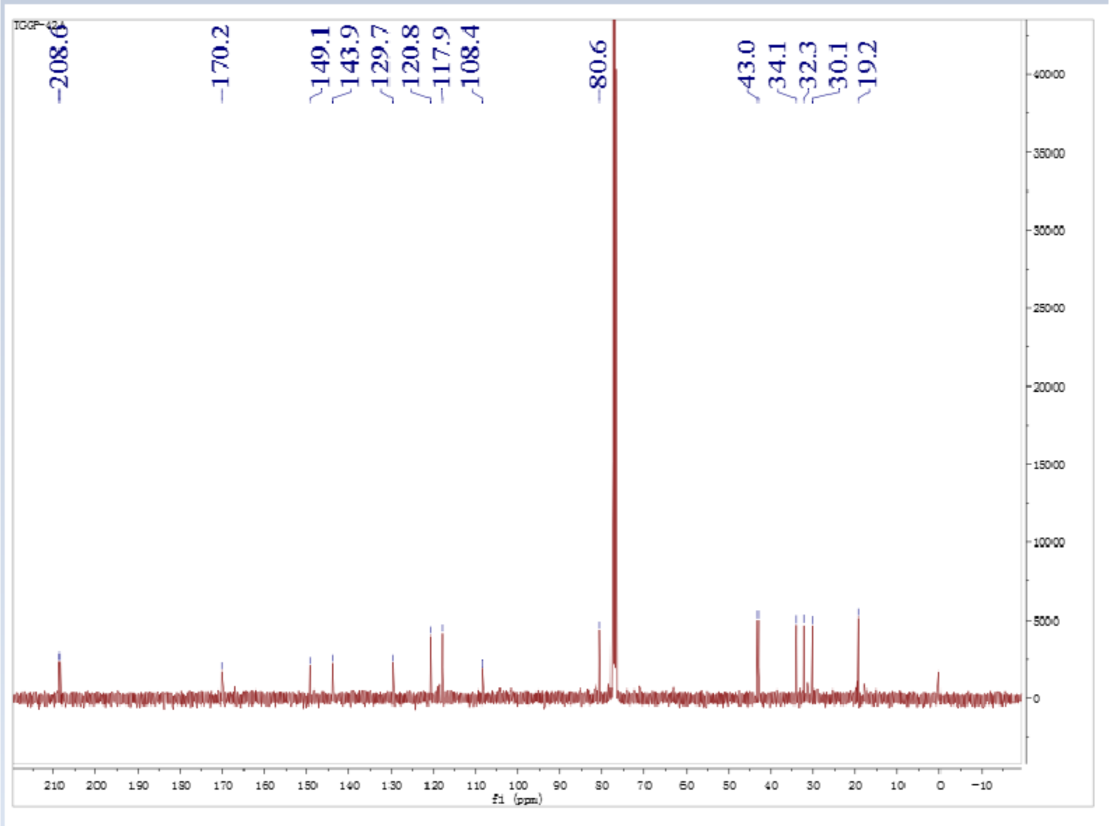

Figure S37. ^{13}C NMR spectrum of **6** in $CDCl_3$

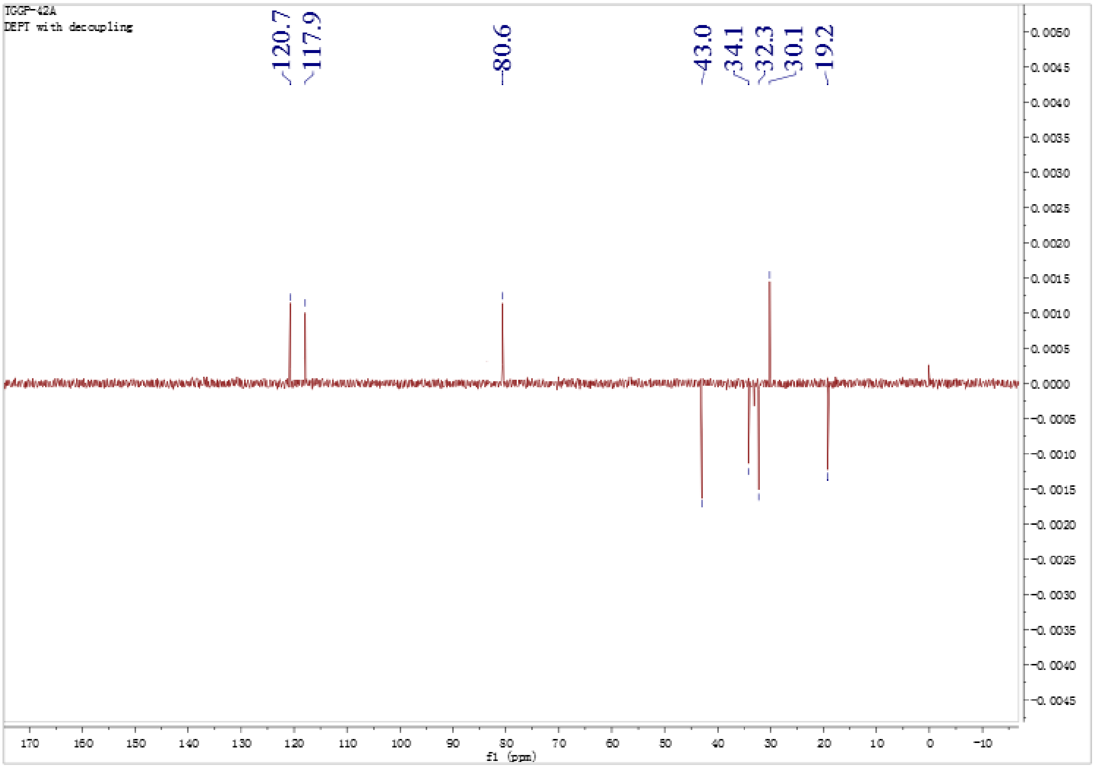

Figure S38. 135-DEPT spectrum of **6** in $CDCl_3$

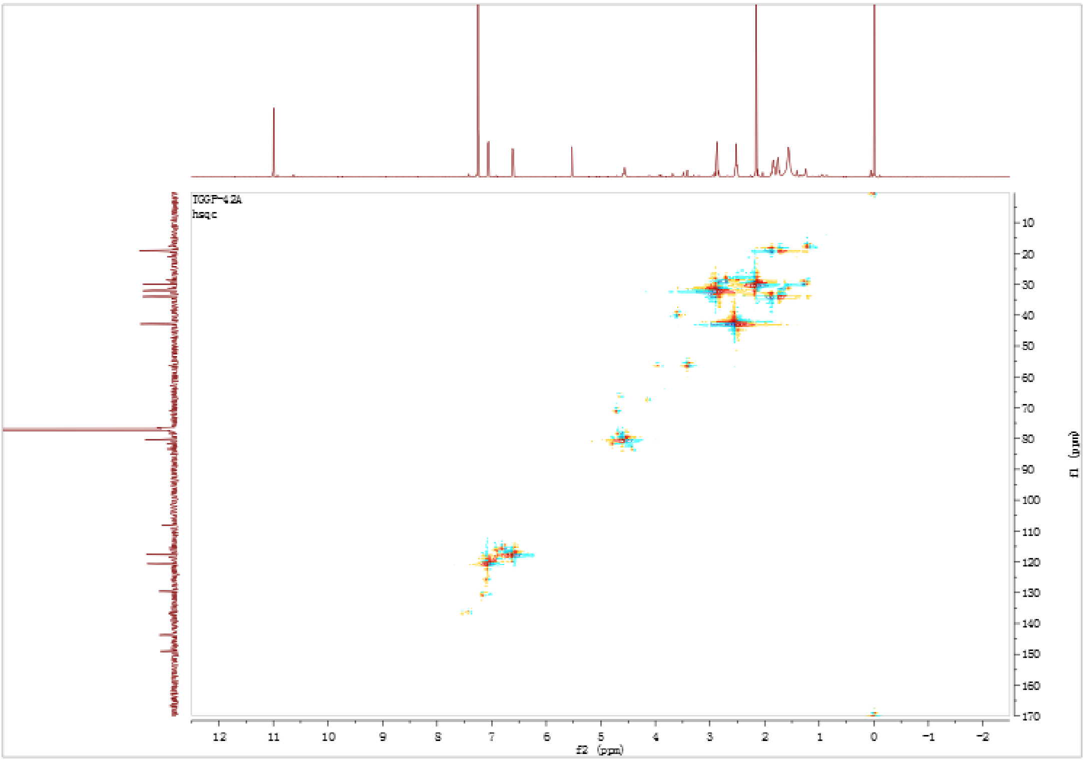

Figure S39. HMQC spectrum of **6** in $CDCl_3$

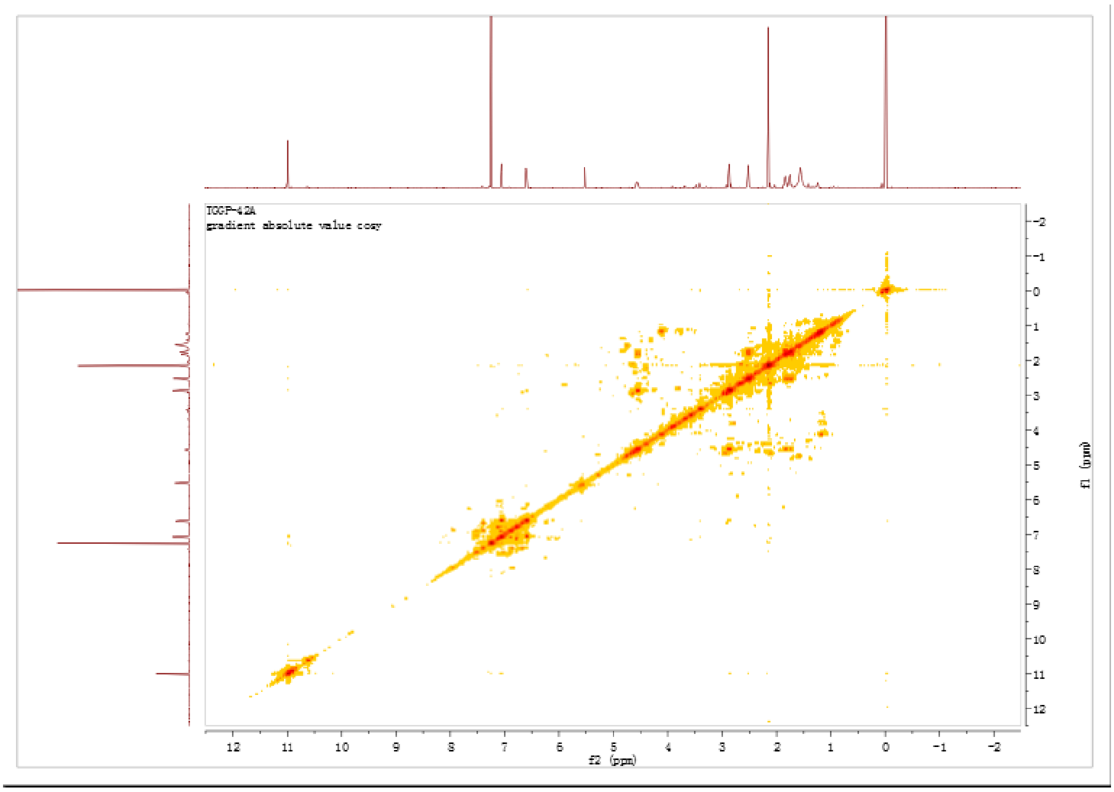

Figure S40. ^{1}H-^{1}H COSY spectrum of **6** in $CDCl_3$

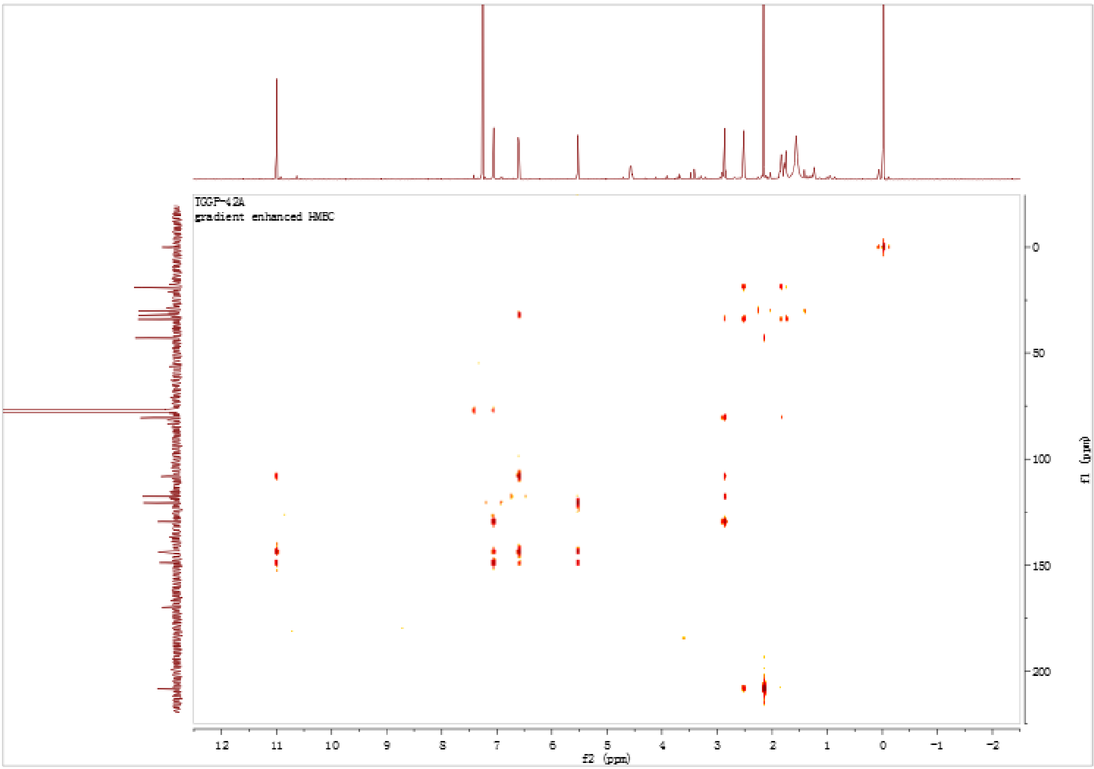

Figure S41. HMBC spectrum of **6** in $CDCl_3$

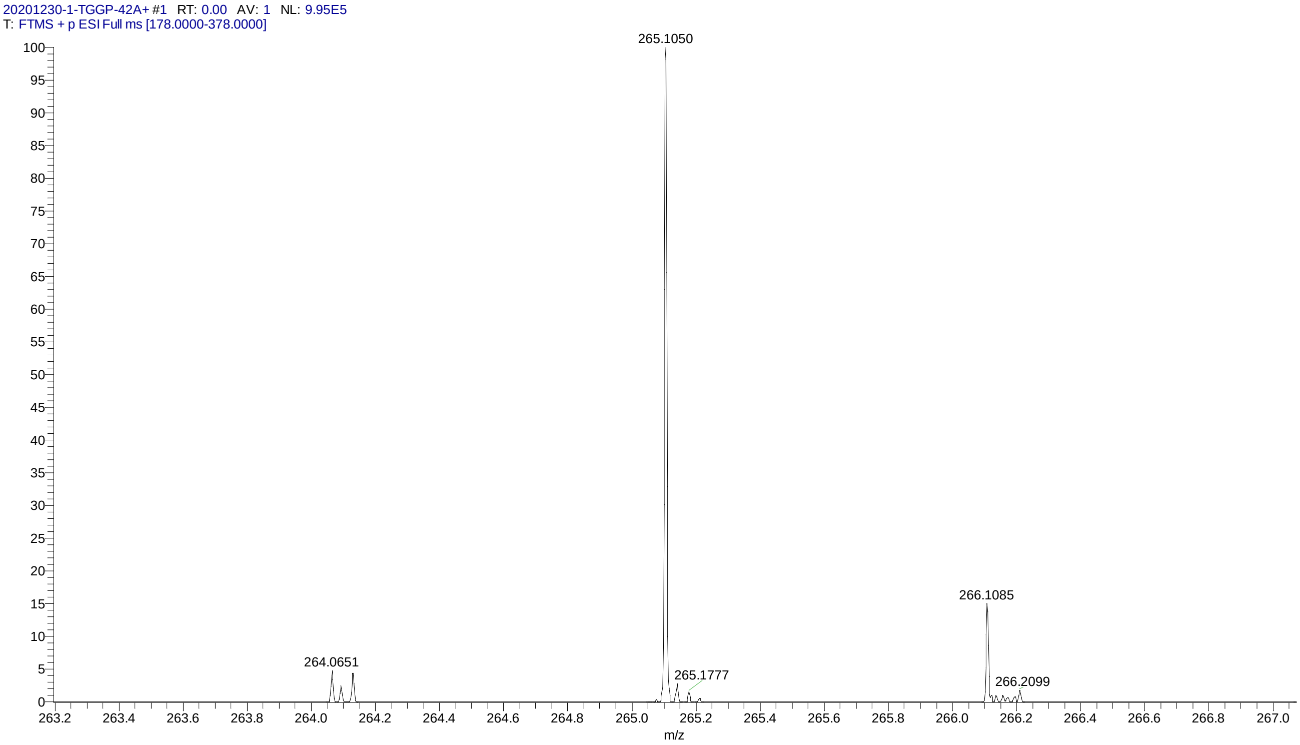

Figure S42. HR-ESI-MS spectrum of **6**

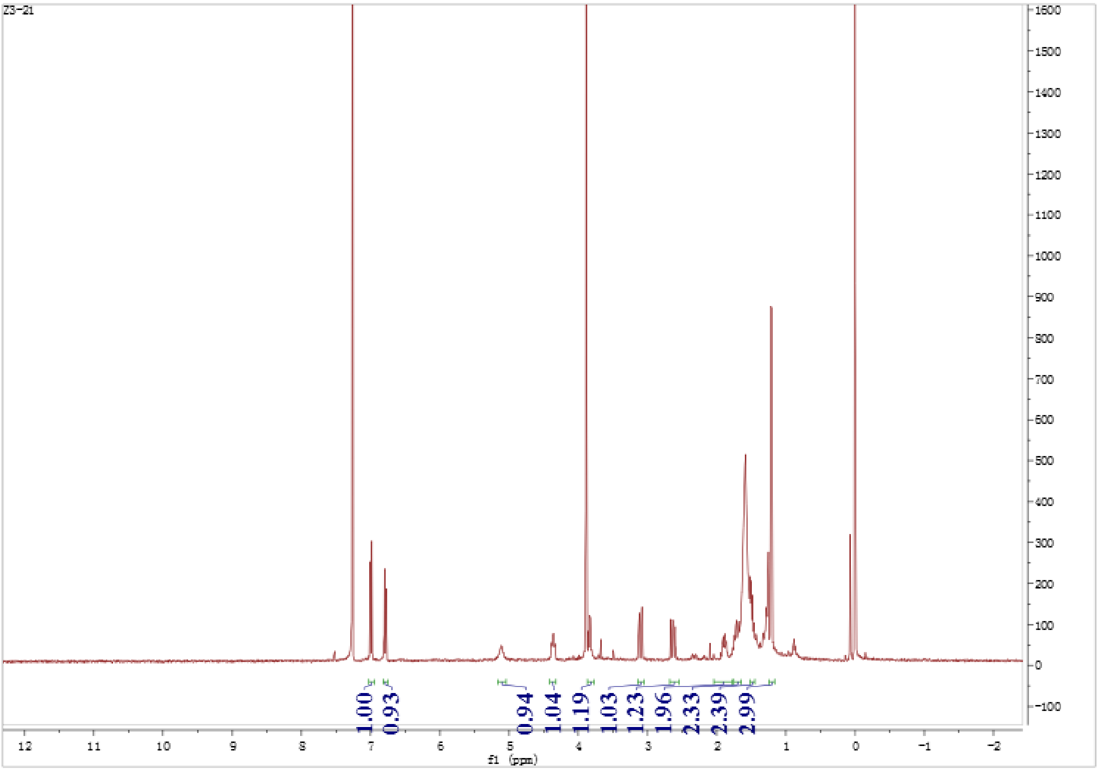

Figure S**43**. ^{1}H NMR ($CDCl_3$, 400 MHz) of Hydrolysate of **1** (**1**a)

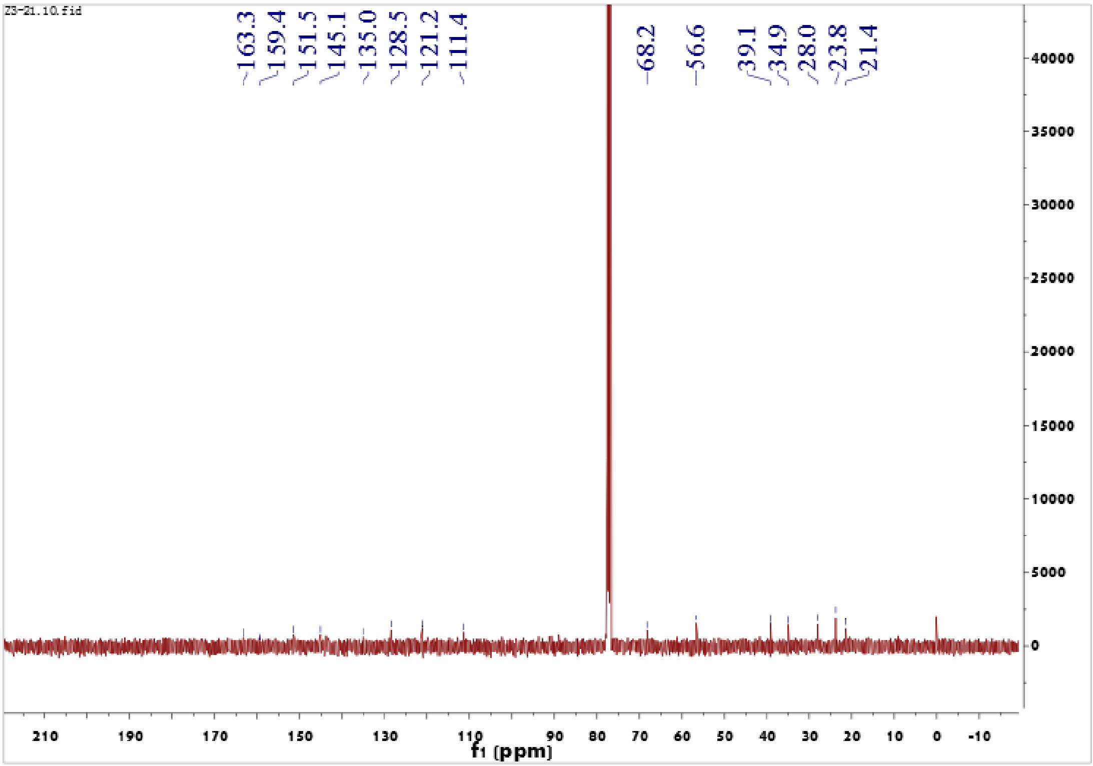

Figure S**44**. ^{13}C NMR ($CDCl_3$, 400 MHz) of Hydrolysate of **1** (**1**a)

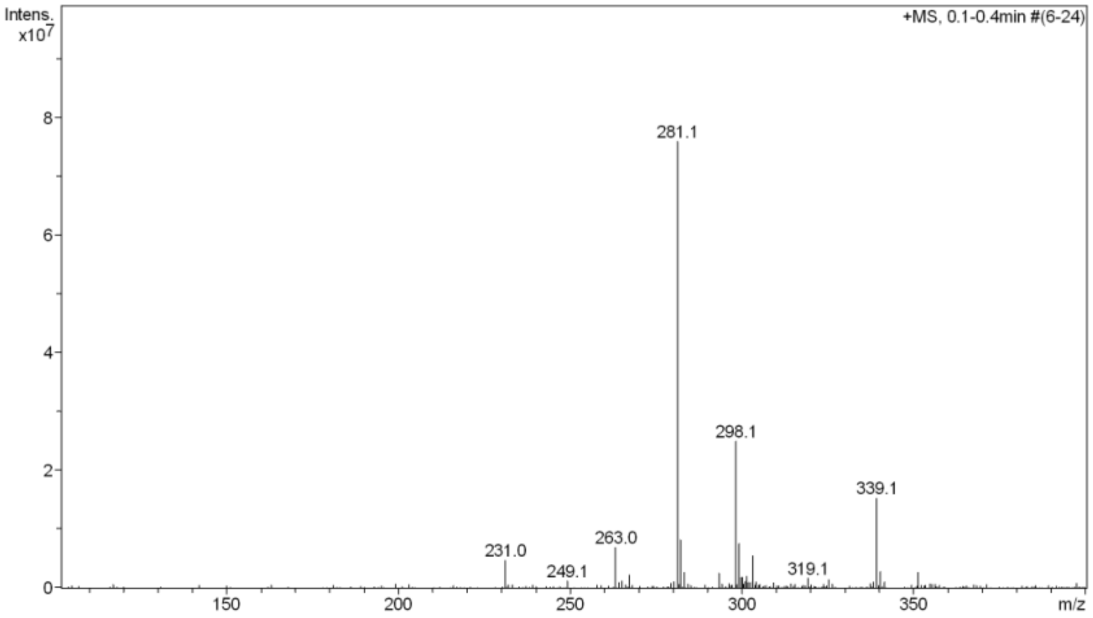

Figure S**45**. ESI-MS spectrum of **1**a

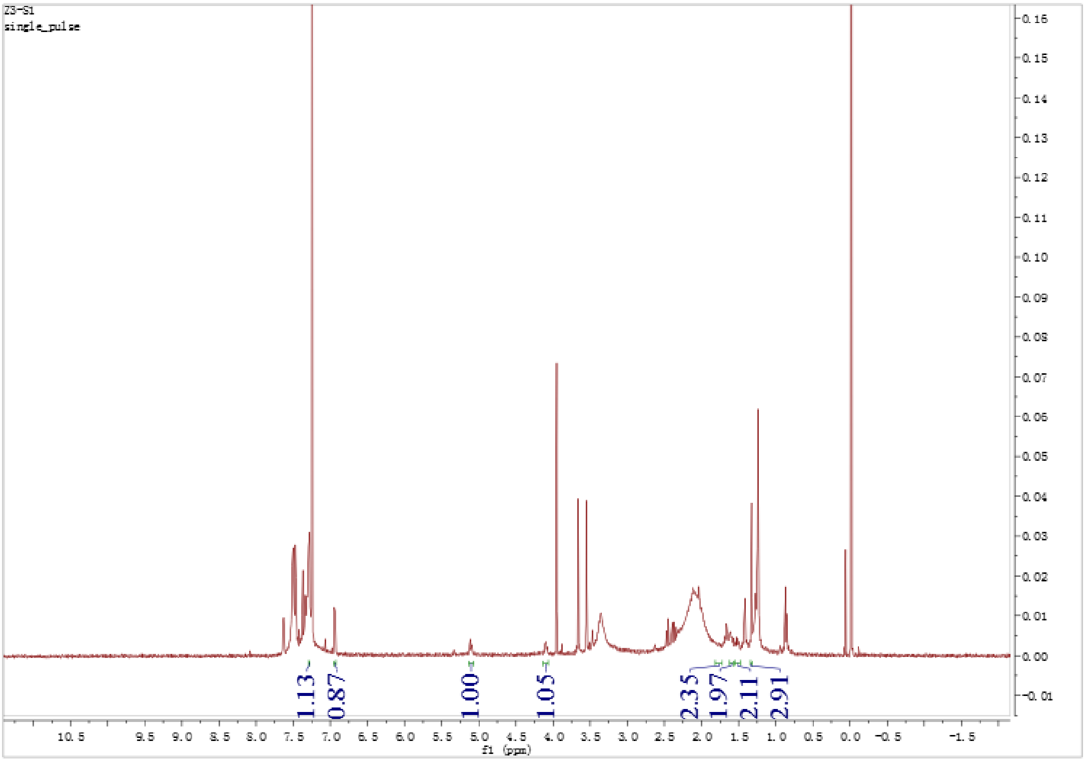

Figure S**46**. ^{1}H NMR ($CDCl_3$, 600 MHz) of *S*-MTPA ester of **1** (**1**b)

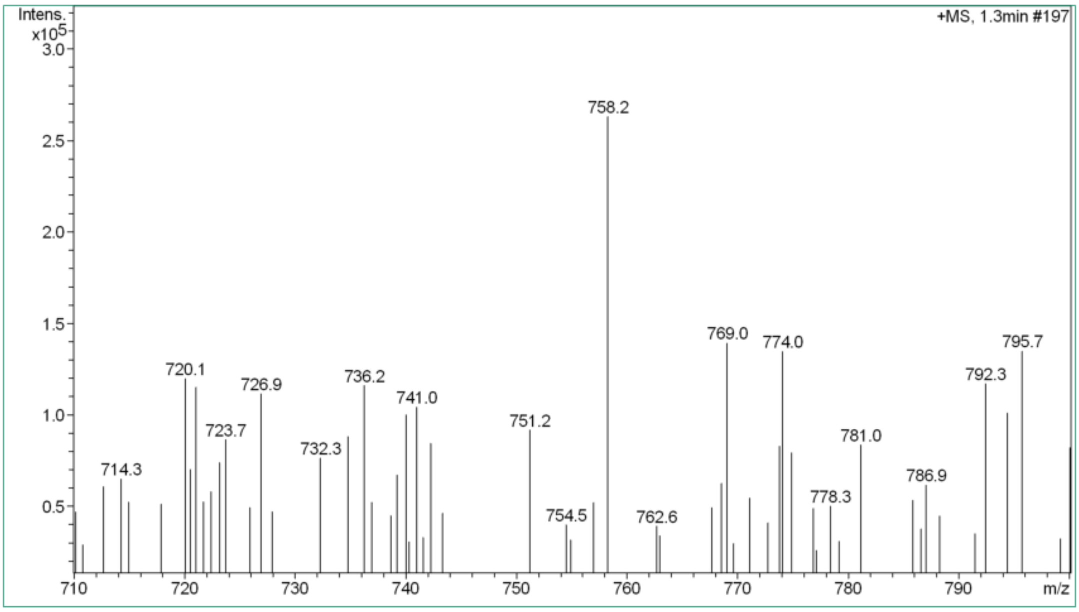

Figure S**47**. ESI-MS spectrum of **1**b

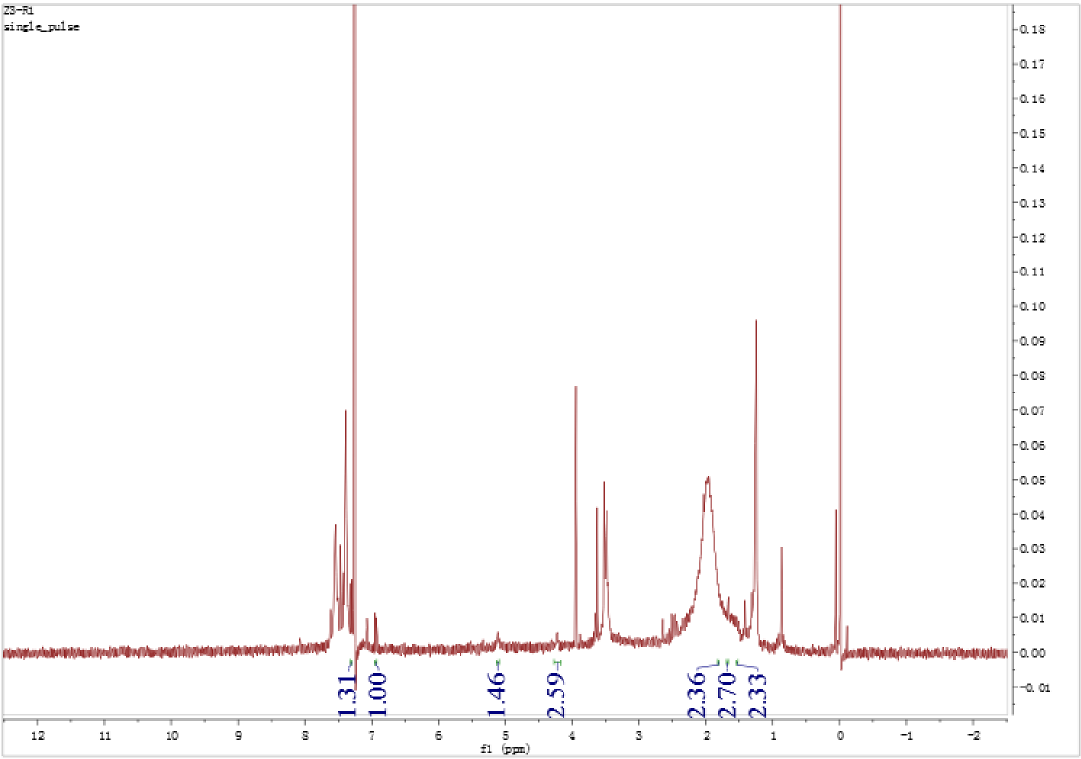

Figure S**48**. ^{1}H NMR ($CDCl_3$, 600 MHz) of *R*-MTPA ester of **1** (**1**c)

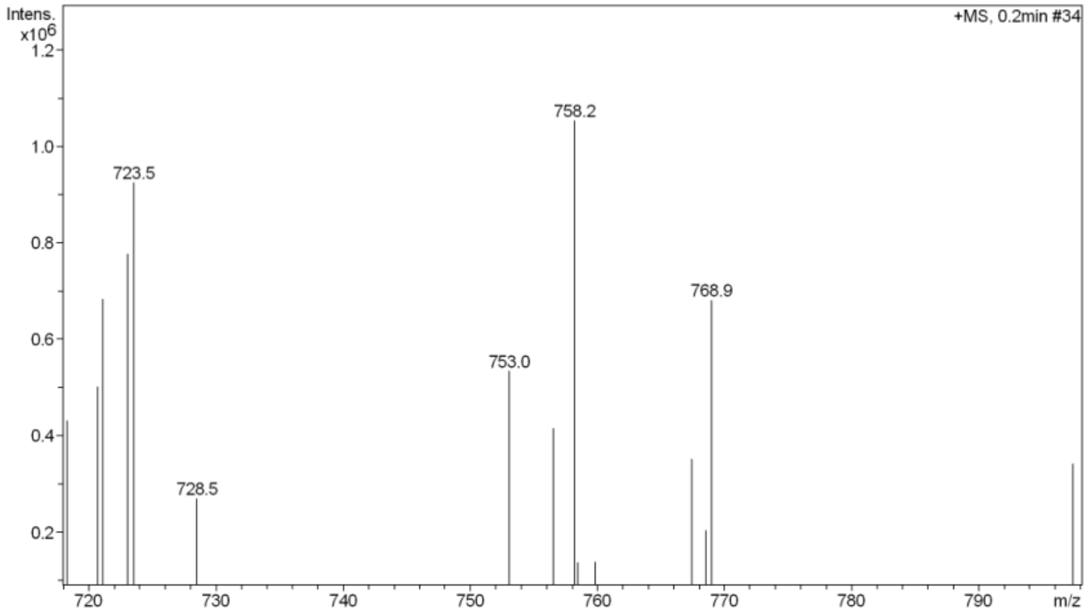

Figure S**49**. ESI-MS spectrum of **1**c

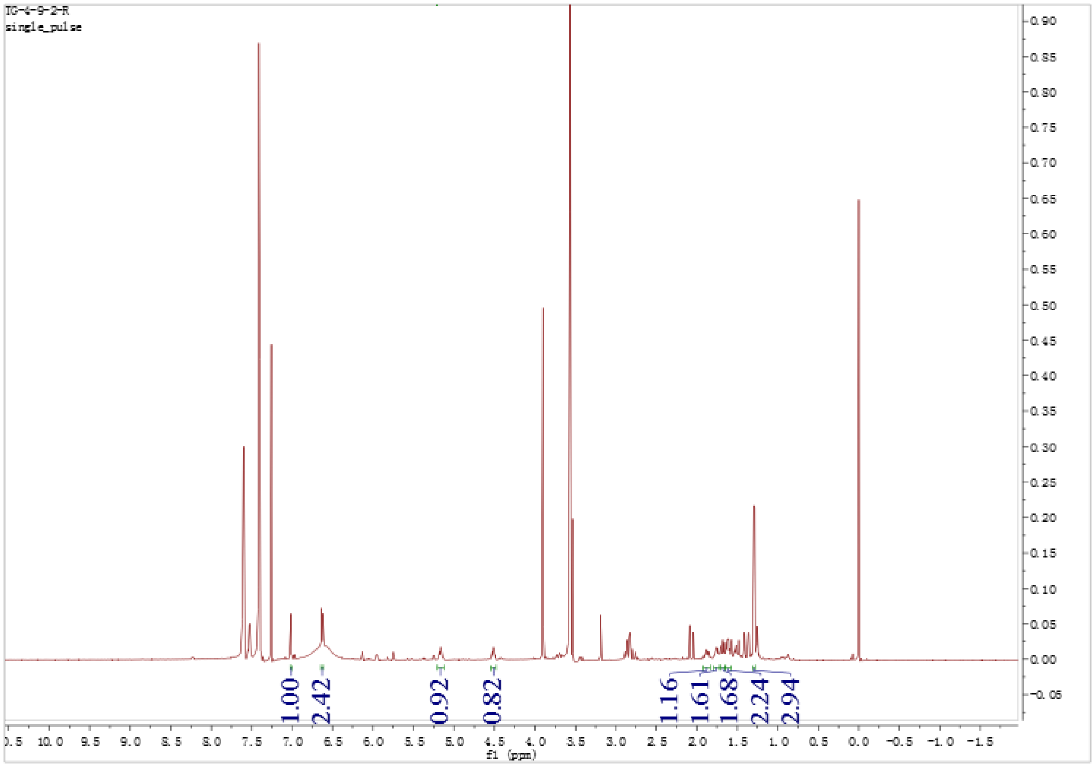

Figure S**50**. ^{1}H NMR ($CDCl_3$, 600 MHz) of *S*-MTPA ester of **2** (**2**a)

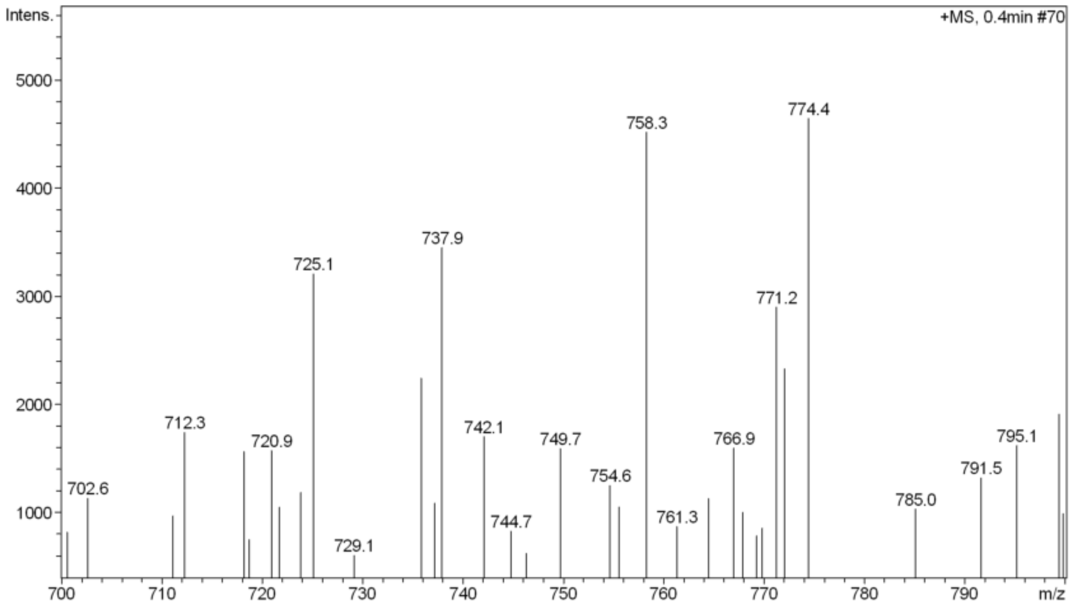

Figure S**51**. ESI-MS spectrum of **2**a

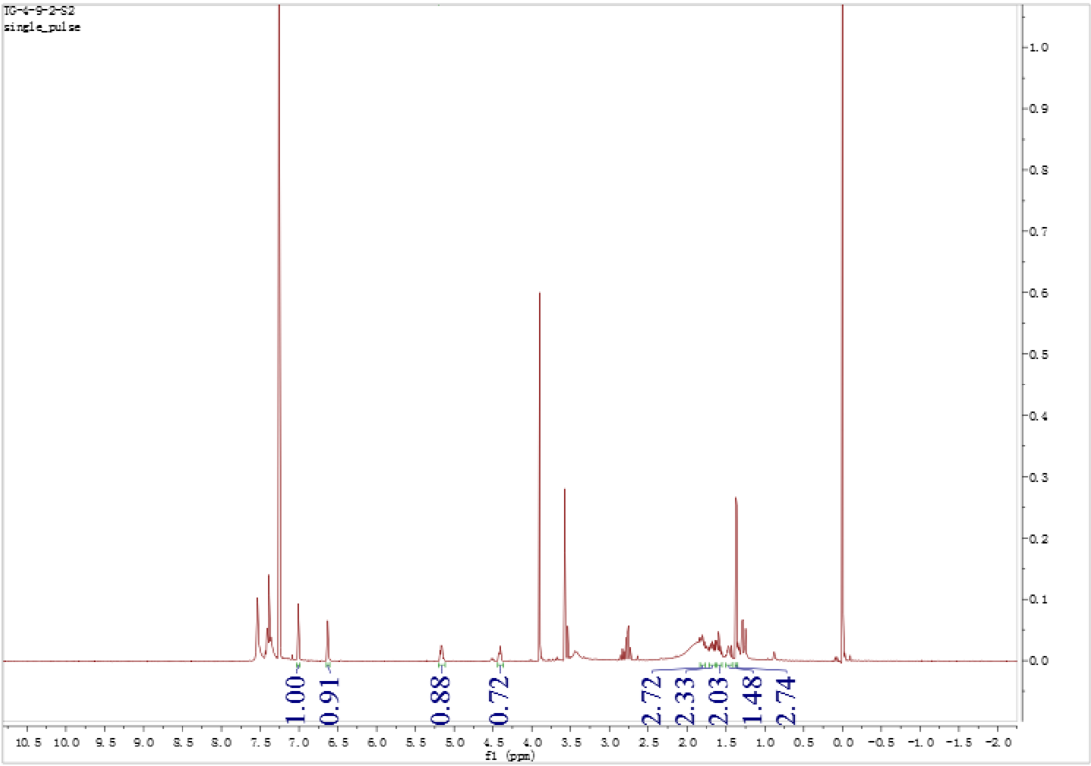

Figure S**52**. ^{1}H NMR ($CDCl_3$, 600 MHz) of *R*-MTPA ester of **2** (**2**b)

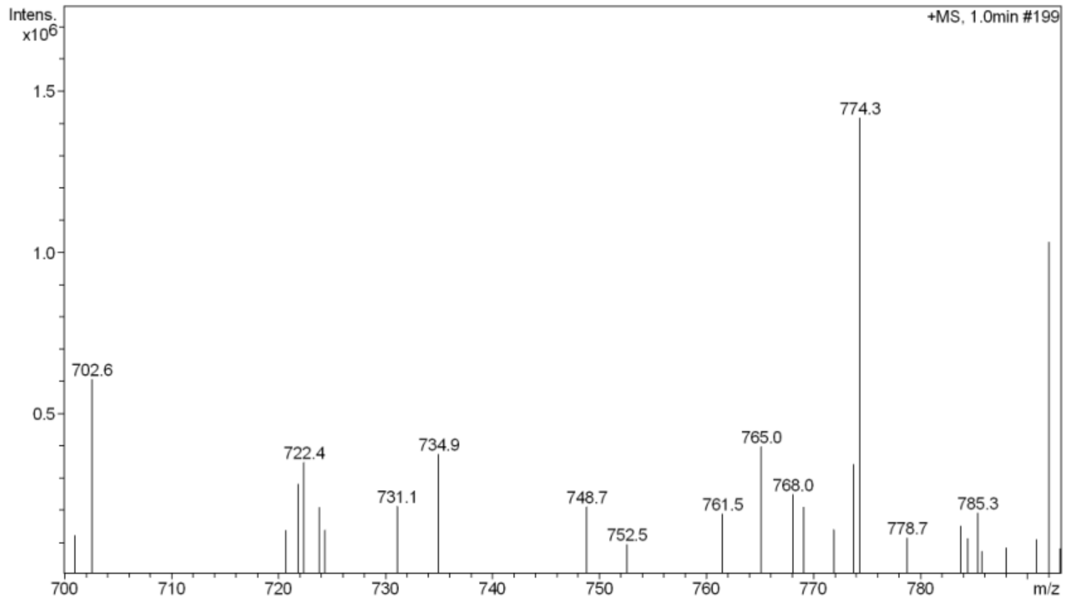

Figure S**53**. ESI-MS spectrum of **2**a

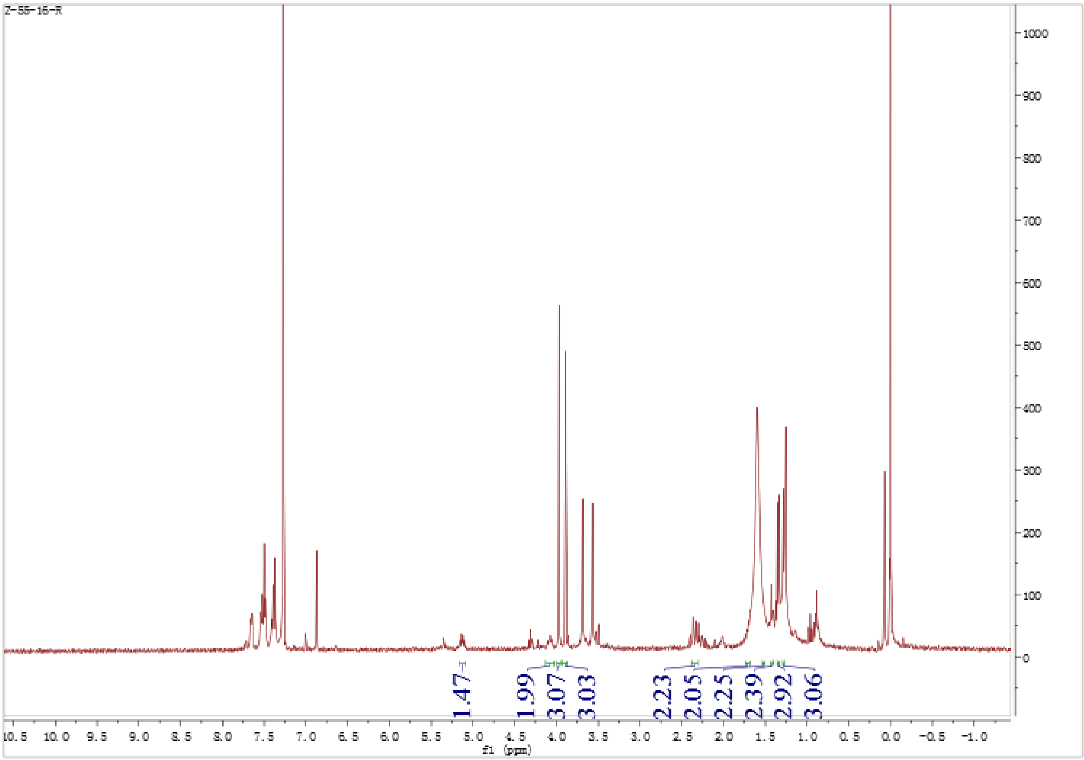

Figure S**54**. ^{1}H NMR ($CDCl_3$, 400 MHz) of *S*-MTPA ester of **3** (**3**a)

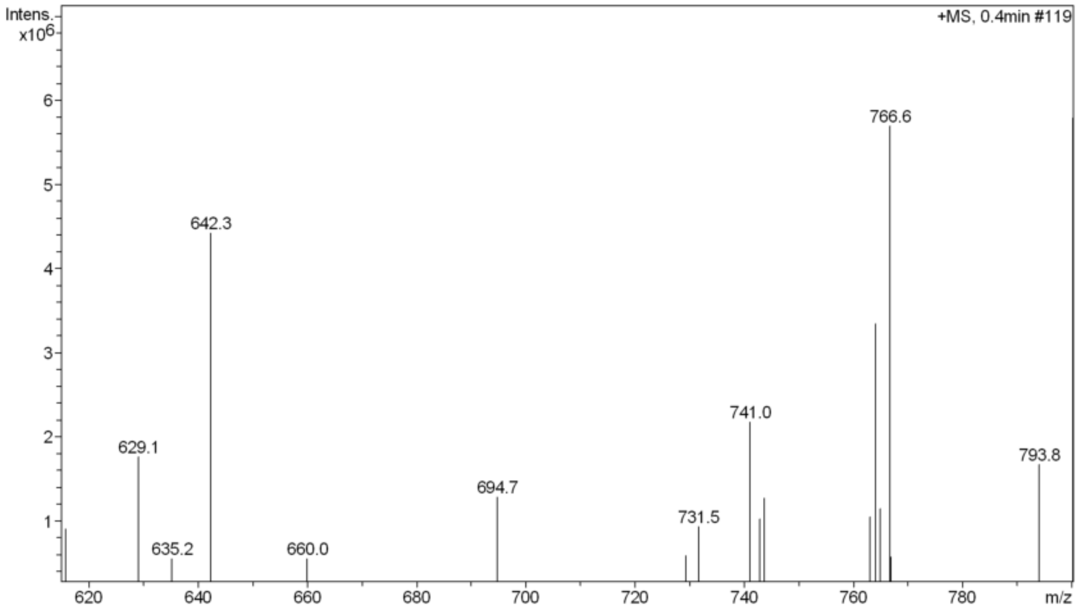

Figure S**55**. ESI-MS spectrum of **3**a

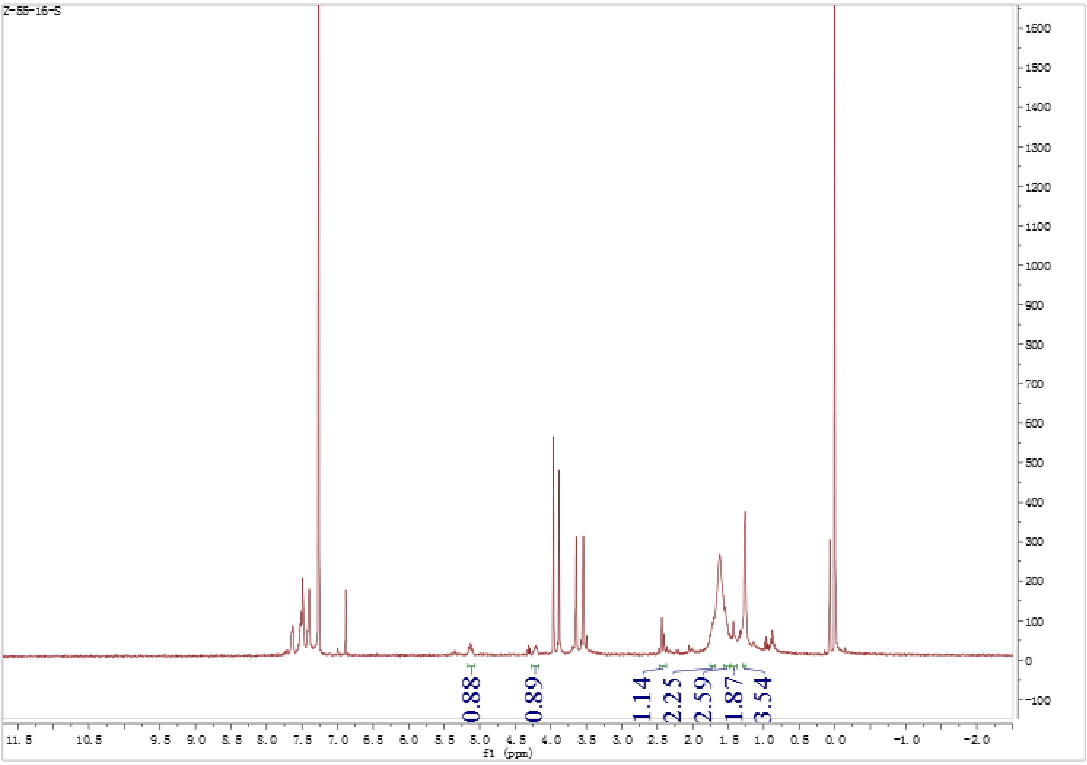

Figure S**56**. ^{1}H NMR ($CDCl_3$, 400 MHz) of *R*-MTPA ester of **3** (**3**b)

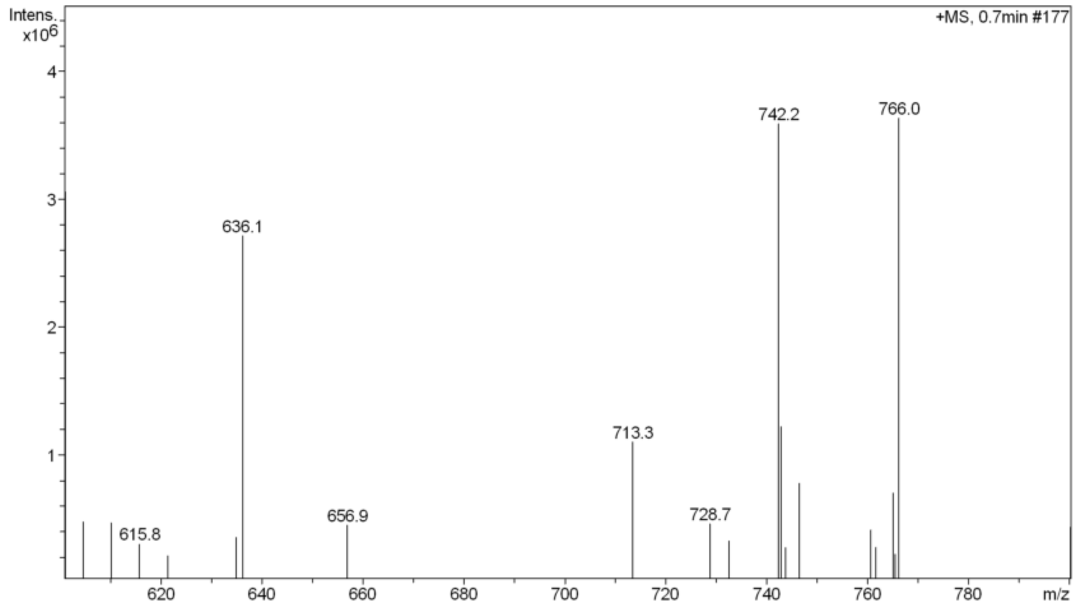

Figure S**57**. ESI-MS spectrum of **3**b
